# The female protective effect in autism spectrum disorder is not mediated by a single genetic locus

**DOI:** 10.1186/s13229-015-0014-3

**Published:** 2015-05-13

**Authors:** Jake Gockley, A Jeremy Willsey, Shan Dong, Joseph D Dougherty, John N Constantino, Stephan J Sanders

**Affiliations:** Department of Genetics, Yale University School of Medicine, 333 Cedar Street, New Haven, CT 06520 USA; Department of Psychiatry, University of California, San Francisco, 401 Parnassus Avenue, San Francisco, CA 94143 USA; Center for Bioinformatics, State Key Laboratory of Protein and Plant Gene Research, School of Life Sciences, Peking University, No.5 Yiheyuan Road, Haidian District, Beijing, 100871 People’s Republic of China; Department of Psychiatry, Washington University, 660 South Euclid Avenue, St Louis, MO 63110 USA; Department of Genetics, Washington University, 4566 Scott Ave, St Louis, MO 63110 USA

**Keywords:** Autism spectrum disorder, Sex bias, Female protective effect, GWAS

## Abstract

**Background:**

A 4:1 male to female sex bias has consistently been observed in autism spectrum disorder (ASD). Epidemiological and genetic studies suggest a female protective effect (FPE) may account for part of this bias; however, the mechanism of such protection is unknown. Quantitative assessment of ASD symptoms using the Social Responsiveness Scale (SRS) shows a bimodal distribution unique to females in multiplex families. This leads to the hypothesis that a single, common genetic locus on chromosome X might mediate the FPE and produce the ASD sex bias. Such a locus would represent a major therapeutic target and is likely to have been missed by conventional genome-wide association study (GWAS) analysis.

**Methods:**

To explore this possibility, we performed an association study in affected *versus* unaffected females, considering three tiers of single nucleotide polymorphisms (SNPs) as follows: 1) regions of chromosome X that escape X-inactivation, 2) all of chromosome X, and 3) genome-wide.

**Results:**

No evidence of a SNP meeting the criteria for a single FPE locus was observed, despite the analysis being well powered to detect this effect.

**Conclusions:**

The results do not support the hypothesis that the FPE is mediated by a single genetic locus; however, this does not exclude the possibility of multiple genetic loci playing a role in the FPE.

**Electronic supplementary material:**

The online version of this article (doi:10.1186/s13229-015-0014-3) contains supplementary material, which is available to authorized users.

## Background

Autism spectrum disorder (ASD) is characterized by impairments in reciprocal social behavior, deficits in language development, and repetitive behavior or restricted interests. ASD is highly heritable [1], and progress has been made in identifying specific genetic loci [2-8] and the pathological mechanisms they target [9-11]. A dramatic sex bias is consistently observed in ASD [12], with males affected more frequently than females. A 4:1 sex bias is frequently cited, with estimates ranging from 2.8:1 to 6.4:1 [13-16]. Several recent publications [2,17-19] have raised the possibility that this sex bias may be the consequence of a female protective effect (FPE) reducing the incidence in females.

The presence of a biological mechanism that reduces the incidence of ASD in a risk-exposed population raises the possibility of artificially inducing this protection as a therapeutic or preventative measure for ASD. Hence, we sought to investigate the molecular nature of the FPE. While the FPE is clearly discordant between sexes, other general sexual dimorphisms could confound discovery of FPE-specific mechanisms. One approach is to try to identify a subset of females in whom the FPE is absent, for example, females with ASD.

Epidemiological evidence suggests that a substantial portion of ASD risk is mediated by genetic risk factors acting in an additive manner [1]. Families with multiple children affected with ASD (multiplex) would be expected to have a higher burden of these genetic risk factors [20], so that the majority of their children would be exposed to high ASD risk. Under this model, we would expect ASD risk to be normally distributed in these children but with a mean risk closer to the ASD diagnostic threshold than in the general population. In females, the FPE results in a higher diagnostic threshold relative to the population mean than in males, leading to a lower female ASD incidence.

**Figure 1:**
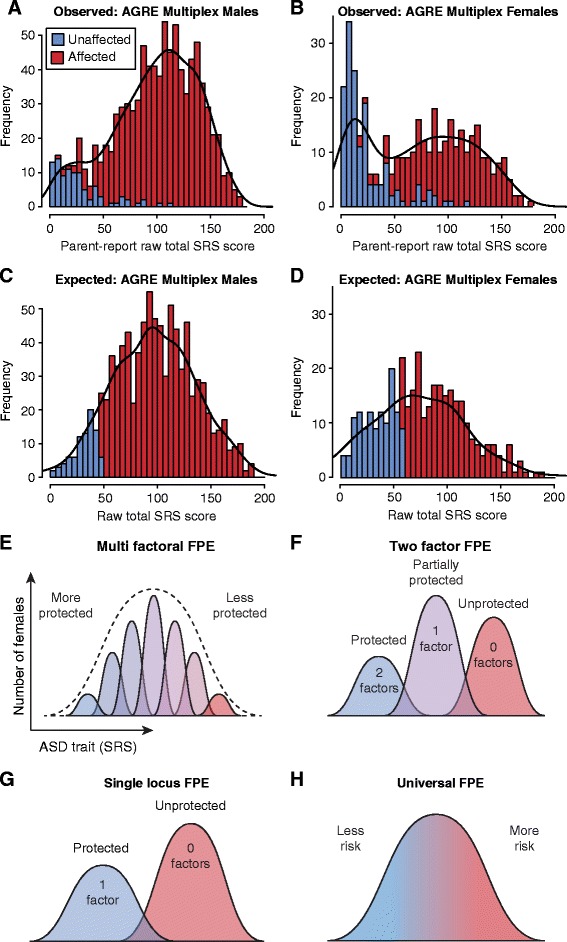
**Expected and observed Social Responsiveness Scale (SRS) scores in multiplex AGRE families.** Children in multiplex families are assumed to have inherited a high degree of ASD risk. Under a threshold model, a quantitative measure of ASD severity, such as the SRS, would be expected to follow a normal distribution with unaffected individuals at the lower end. **(A)** The observed SRS scores for 927 male children (95 unaffected in blue, 832 affected in red) with each bar showing the sum of the number of unaffected and affected males. The black line shows the kernel density of the data, which approximates a normal distribution. **(B)** The corresponding plot is shown for 394 female children (151 unaffected, 243 affected). The SRS scores produce a bimodal distribution, as noted previously [22,23]. **(C)** To assess the expected distribution under quantitative trait model, we estimated the mean and standard deviation of the male observed data ‘A’ and used these characteristics to simulate a normal distribution for the same number of individuals. The scores were sorted, and a threshold for affected status was chosen to give the same number of affected and unaffected males as in ‘A’. Each bar shows the sum of the number of unaffected and affected simulated males, while the black line shows the kernel density. **(D)** The expected distribution under quantitative trait model is shown using the same method as in ‘C’ but for 394 females based on the female data in ‘B’. The expected distribution differs markedly from the observed in females, but not in males. **(E)** If multiple factors contribute to the presence of the FPE, then their combined effect is likely to produce a unimodal distribution. **(F)** As the number of factors contributing to the presence of the FPE decreases, the unimodal distribution in ‘E’ develops distinct distributions based on the number of factors present. **(G)** If only one factor contributes, then a bimodal distribution should be observed. **(H)** Finally, if there are no factors and the FPE is universally present in females, a unimodal distribution will arise based on the distribution of risk rather than protection.

The Social Responsiveness Scale (SRS) is a quantitative measure of ASD behaviors in affected and unaffected individuals [21]. Treating the SRS as a proxy for the underlying ASD risk, we would expect the SRS to be normally distributed in the children of multiplex families, with a higher diagnostic threshold relative to the population mean in females than in males. The observed distribution in males from multiplex families (Figure 1A) approximates this expectation (Figure 1C); however, females in multiplex families show a bimodal distribution (Figure 1B) that differs from expectation (Figure 1D). This bimodal distribution has been reported in ASD cohorts from the Autism Genetic Resource Exchange (AGRE) and the Interactive Autism Network (IAN) [22,23]. There is a substantial difference of about 90 SRS points (4.5 standard deviations) between the two peaks of the bimodal distribution in Figure 1B, suggesting distinct subsets within the female cohort. In contrast, female SRS scores follow a unimodal distribution in the general population with a mean score 3 points (0.17 standard deviations) lower than for general population males [24-26].

We considered whether this bimodal distribution could reflect the categorical presence (low score) or absence (high score) of a protective effect in females. If the FPE was, itself, mediated by multiple protective factors, we would still expect a normal distribution of SRS scores (Figure 1E) with the mean shifted towards lower SRS scores compared with males (Figure 1C,D). As the number of protective factors decreases, distinct distributions would be expected based on the presence or absence of the factors (Figure 1F) with a single protective factor mediating the FPE leading to a bimodal distribution (Figure 1G), as observed (Figure 1B). This leads to the hypothesis that a single common genetic variant is responsible for the FPE (Additional file 1: Figure S1).

**Figure 2:**
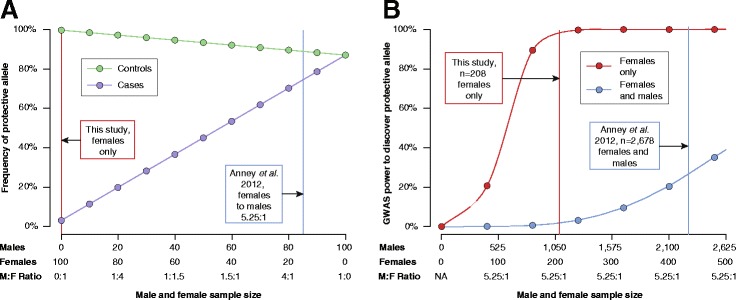
**GWAS power estimate for a single factor mediating the FPE. (A)** In females exposed to high ASD risk, the protective factor will be enriched in unaffected individuals (green) and largely absent in cases (purple). We estimate a distinct difference in the frequency of the protective allele in these two cohorts (Additional file 1: Supplementary Methods) for an analysis based only on females (red line). Conversely, the protective allele has no effect in males and will be observed at an equal frequency in male cases and controls. Including males in a GWAS analysis will therefore add noise (blue line, representing the observed 5.25:1 ratio of males to females in Anney *et al.* [27]) resulting in a reduction in power. **(B)** An estimate of GWAS power to detect a single FPE allele in females only (red) and females and males (blue) under a model where protection contributes 50% of the observed 5.25:1 sex bias. The vertical lines represent the sample size in this study (red) and the Anney *et al.* [27] GWAS study (blue).

To estimate whether a genome-wide association study (GWAS) would detect such a protective factor, we performed a power analysis. We expect the protective factor to be enriched in female controls compared with female cases, but to have no effect in male subjects, therefore the presence of males adds ‘noise’ to a GWAS analysis (Figure 2A). Under ‘ideal’ conditions, that is, that the 4:1 sex bias was solely the consequence of the FPE and that the FPE was sufficient to prevent an ASD diagnosis, we found that the largest GWAS to date (2,678 cases and 2,678 pseudocontrols [27]) would have 100% power to detect such a protective allele. However, an assumption of ideal conditions is unlikely to be accurate. Therefore, we estimated the power if the FPE was only responsible for 50% of the observed 4:1 sex bias as a means to model deviation from ideal conditions (Figure 2B). The power was reduced to 30% (Additional file 1: Supplementary Methods and Figures 2B and Additional file 1: Figure S2). We then repeated this power estimate for a GWAS performed only on the females, who represented 16% (5.25:1) of the cases [27], and found that the power would increase from 30% to 100% (Figure 2B). In fact, by varying the cohort size, we found that a female subject GWAS dramatically increased the power across a wide range of conditions (Additional file 1: Figure S2). We therefore concluded 1) that the GWAS conducted so far would probably have missed a single locus FPE and 2) that a female-only GWAS would be very well powered to find such an effect across a wide range of assumptions.

**Figure 3:**
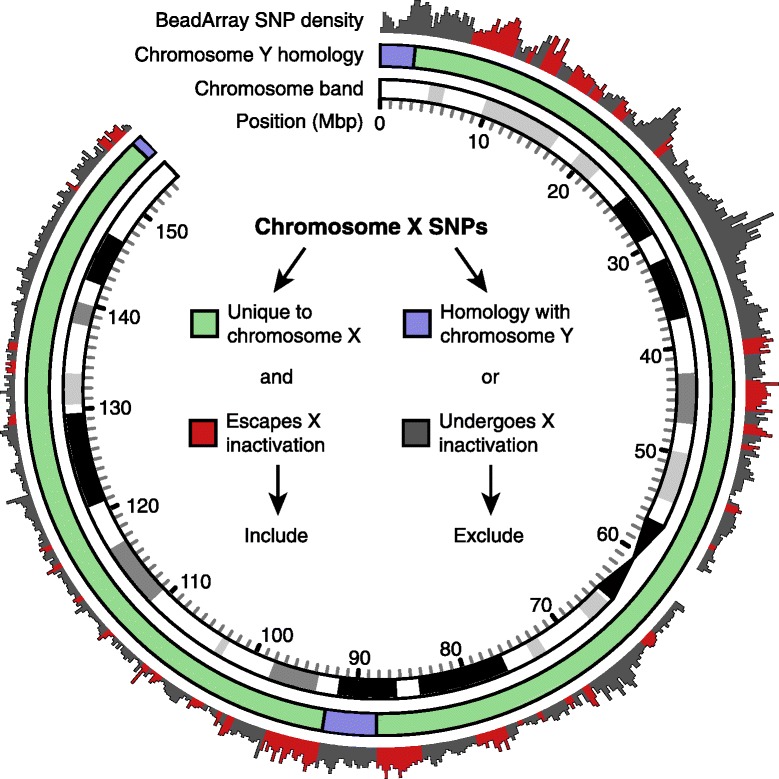
**Identification of chromosome X SNPs that escape X-inactivation for tier 1 analysis.** This Circos plot shows the length of chromosome X proceeding clockwise with position 0 on the short arm at twelve o’clock. Adjacent to the chromosome position, the innermost ring indicates chromosome banding by the depth of shading; two opposing black arrows indicate the centromere. Regions of chromosome Y homology are shown in purple in the middle ring; SNPs in these regions were excluded from the tier 1 analysis leaving the SNPs unique to chromosome X indicated in green. The outermost ring shows SNP density based on the genotyping array (see ‘Methods’ section) by the height of the bars. Regions that are inactivated on one copy of chromosome X are shown in gray [32] and SNPs in these regions were excluded from the tier 1 analysis, leaving only SNPs that escape X-inactivation, shown in red (Additional file 2: Table S4). Of the 6,955 SNPs on chromosome X, 451 (6.5%) were included in the tier 1 analysis.

Based on these results, we performed a GWAS on the AGRE dataset, comparing 208 affected females with 151 unrelated unaffected females. To maximize our power, we considered single nucleotide polymorphisms (SNPs) in three tiers as follows: 1) SNPs unique to chromosome X that escape X-inactivation (Figure 3 and Additional file 2: Table S4), since the increased dosage in females provides a simple mechanism for female-specific protection; 2) All SNPs on chromosome X; and 3) All SNPs across the whole genome. We used 207 affected females and 676 unrelated unaffected females from the Simons Simplex Collection (SSC) as a replication set. The SSC was not used for discovery since affected status is less likely to be determined by FPE absence, due to the lower contribution of inherited risk [27] and higher contribution of *de novo* risk [5-7,18,19] in simplex families.

While the presence of a single locus mediating the FPE may seem unlikely, the potential therapeutic implications of such a finding are so great that it was important to fully explore this possibility. To our knowledge, no previous molecular genetic study of autism has reported the results of such an analysis.

## Methods

### Subjects and genotyping

Genotyping data were collated from two independent large cohorts of ASD families: 1,976 families from the AGRE [28] and 2,733 families from the SSC [13].

The AGRE data were generated on one of the three Illumina BeadArrays (Illumina, Inc., San Diego, CA, USA): 550v1 (421 families), 550v3 (1,277 families), and Omni 1M (278 families). Analysis was restricted to the 329,483 SNPs shared between all three arrays. The SSC data were generated on one of the three Illumina BeadArrays: 1Mv1 (421 families), 1Mv3 Duo (1,277 families), and Omni 2.5M (1,035 families). Analysis was restricted to the 493,924 SNPs shared between all three arrays.

### Ancestry and data cleaning

Data were restricted to families of European ancestry, and standard GWAS data cleaning were performed. European ancestry was determined using EIGENSTRAT [29] and the four core HapMap populations [30] (Additional file 1: Figure S3). The resulting genomic inflation for European samples was 1.03 (Additional file 1: Figure S3). SNP data were cleaned using PLINK [31], specifically we only included SNPs with minor allele frequency ≥0.03 (Additional file 1: Supplementary Methods), genotype rate of ≥0.95 per sample (minimum observed genotyping rate was 0.991), genotype missingness per SNP ≤0.1, and Hardy-Weinberg equilibrium <0.0001.

After data cleaning, there were 943 families and 317,574 SNPs for AGRE and 2,166 families and 440,778 SNPs for SSC.

### Identifying unrelated females

Of the 943 remaining AGRE families, only 510 contained at least one female with genotyping data. Where a family had multiple females, only one was selected, with a preference for unaffected females, since these are less frequent in the AGRE sample. From these, 151 unaffected females and 208 affected females (defined as ‘autism’ or ‘broad spectrum’) were identified and used for the analysis. Identity by descent demonstrated that these samples were all unrelated (Additional file 1: Figure S4).

A similar approach was applied to the 2,166 remaining SSC families, of which 883 had at least one female. In families with multiple females, only one was selected, with a preference for affected females, since these are less frequent in the SSC sample. The analysis was therefore performed on 207 affected females and 676 unaffected females. A complete list of the samples included in the analysis can be found in Additional file 3.

### Determining SNPs of interest

For the first tier of analysis, SNPs on chromosome X were selected if they lacked homology to chromosome Y and escaped X-inactivation (Figure 3 and Additional file 2: Table S4) [32]. These regions represent 14% of chromosome X (21.8 Mbp). This left 451 SNPs for analysis in the AGRE data and 720 SNPs in the SSC data. For the second tier analysis, all of chromosome X was considered with 6,955 SNPs in AGRE and 10,269 SNPs in SSC. Finally, for the third tier of analysis, all SNPs that remained after cleaning were included with 317,574 SNPs in AGRE and 440,778 SNPs in SSC.

### Association testing

Association tests were performed using PLINK [31] under a dominant model. All *P* values were corrected for multiple comparisons, using Bonferroni correction based on the number of SNPs analyzed in each tier. The cluster plots of all SNPs highlighted by the analysis are shown in Additional file 1: Figures S14 to S17.

### Power calculation

Power was estimated using G*Power 3.1 [33], based on the Fisher exact test. Hypothesized allele frequencies in cases and controls were derived from the 4:1 sex bias (see Additional file 1: Supplementary Methods). An alpha of 0.05 after Bonferroni correction (based on the number of SNPs analyzed) was used.

## Results

### Targeted association study: tier 1 SNPs

**Figure 4:**
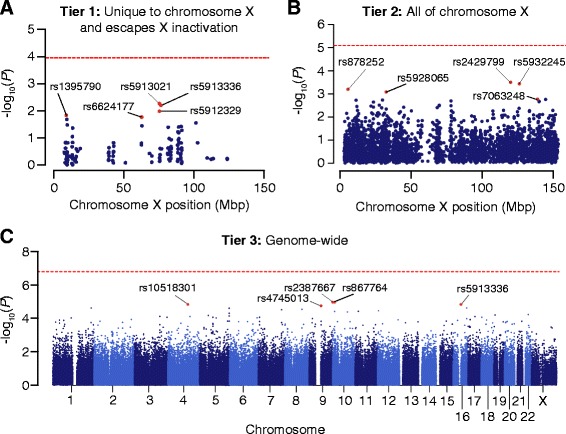
**Manhattan plots of association study results.** Results of association studies comparing 208 affected females and 151 unaffected females from AGRE. To maximize the ability to identify a candidate variant for the FPE the association test was performed on three tiers of SNPs, based on the *a priori* probability of mediating the FPE. **(A)** Tier 1: 451 SNPs unique to chromosome X that escape X-inactivation. No SNPs are significant after multiple comparisons (horizontal red line). The top five SNPs (red) are labeled (Table 1). **(B)** Tier 2: all 6,955 SNPs on chromosome X. No SNPs are significant after multiple comparisons (horizontal red line). The top five SNPs (red) are labeled (Table 2). **(C)** Tier 3: all 317,574 SNPs across the genome. No SNPs are significant after multiple comparisons (horizontal red line). The top five SNPs (red) are labeled (Table 3).

To test the hypothesis that the FPE is mediated by a common variant at a single locus, we performed an association test comparing 208 affected females against 151 unrelated unaffected females. Since the FPE is unique to females, we reasoned that the region of the genome that has the greatest potential for sexual dimorphism would be the most likely location for such a locus, and therefore, the first tier of our analysis was performed on 451 SNPs that are unique to chromosome X and that escape X-inactivation (Figure 3 and Additional file 3: Table S4). No SNPs were significant after correcting for the 451 comparisons (Figure 4A). Of the top five SNPs (Table 1), only two had a dominant risk allele that was observed more frequently in the affected females (odds ratio >1) and none had allele frequencies close to the prediction in both the affected and unaffected groups (Additional file 1: Supplementary Methods). Only one of these five SNPs was represented on the microarrays used for the SSC replication cohort (207 affected females, 676 unaffected females); despite this SNP reaching nominal significance, the dominant risk allele was more frequent in the affected group, that is, the opposite direction of effect observed in the discovery sample. Given the targeted nature of this analysis, the estimated power to discover a single locus meeting our hypothesis was 100% even with modest enrichment of unprotected females in the affected group (Additional file 1: Figure S1).

**Table 1:** **Top five SNPs from tier 1 analysis: unique to chromosome X in regions that escape X-inactivation**

	**AGRE discovery (208 cases, 151 controls)**						**SSC replication (207 cases, 676 controls)**			
**SNP**	**Allele 1/allele 2**	**Affected allele 1 frequency (%)**	**Unaffected allele 1 frequency (%)**	**Odds ratio**	***P*** **value**	**Corrected** ***P*** **value**	**Affected allele 1 frequency (%)**	**Unaffected allele 1 frequency (%)**	**Odds ratio**	***P*** **value**
Predicted	.	97.0	<24.3	>88	≤1×10^−8^	≤0.05	97.0	<24.3	>88	≤1×10^−8^
rs5913021	G/A	71.5	57.3	1.25	0.005	1.0	.	.	.	.
rs5913336	A/G	49.0	63.6	0.77	0.006	1.0	69.6	60.9	1.14	0.007
rs5912329	A/G	71.4	58.2	1.23	0.01	1.0	.	.	.	.
rs1395790	G/A	2.4	7.9	0.30	0.01	1.0	.	.	.	.
rs6624177	A/G	60.0	72.2	0.83	0.02	1.0	.	.	.	.

### Targeted association study: tier 2 SNPs

Since no clear candidates were observed in the tier 1 SNPs, we expanded the analysis to the whole of chromosome X to account for the possibility that our knowledge of regions escaping X-inactivation may not be complete.

As with the tier 1 analysis, no SNPs showed significant association after correcting for the 6,955 comparisons (Figure 4B). Considering the top SNPs (Table 2), all five showed a direction of effect that was consistent with expectation, but with a lower odds ratio (see Additional file 1: Supplemental Methods). None of these SNPs were nominally significant in the SSC replication cohort. Of note, none of the top five SNPs from the tier 1 analysis were in the top five for the tier 2 analysis, despite all 451 tier 1 SNPs being included in this analysis. We estimated our power to detect the hypothesized single FPE locus to still be 100% for tier 2.

**Table 2:** **Top five SNPs from tier 2 analysis: all chromosome X SNPs**

	**AGRE discovery (208 cases, 151 controls)**						**SSC replication (207 cases, 676 controls)**			
**SNP**	**Allele 1/allele 2**	**Affected allele 1 frequency (%)**	**Unaffected allele 1 frequency (%)**	**Odds ratio**	***P*** **value**	**Corrected** ***P*** **value**	**Affected allele 1 frequency (%)**	**Unaffected allele 1 frequency (%)**	**Odds ratio**	***P*** **value**
Predicted	.	97.0	<24.3	>88	≤1×10^−8^	≤0.05	97.0	<24.3	>88	≤1×10^−8^
rs2429799	A/G	21.2	7.3	2.90	0.0003	1.0	.	.	.	.
rs5932245	A/G	97.1	87.4	1.11	0.0004	1.0	54.1	55.0	0.98	0.9645
rs878252	G/A	31.7	15.9	2.00	0.0006	1.0	29.0	25.6	1.13	0.1699
rs5928065	G/A	94.7	84.1	1.13	0.0008	1.0	88.4	93.3	0.95	0.5674
rs7063248	G/A	73.4	57.6	1.27	0.0017	1.0	97.1	96.6	1.01	0.1885

### Genome-wide association study: tier 3 SNPs

Next, we considered the possibility that the protective allele was not on chromosome X, (for example, an autosomal gene that was only expressed in the presence of high estrogen levels). We therefore repeated the analysis for all 317,574 SNPs in the AGRE group. Again, there was no association after correction for multiple comparisons (Figure 4C), and none of the top five SNPs were nominally significant in the replication group (Table 3). Of note, none of the top five SNPs were on chromosome X. Even with the larger number of SNPs, we estimated our power to detect the hypothesized single FPE locus to be 100% (Additional file 1: Figure S2).

**Table 3:** **Top five SNPs from tier 3 analysis: genome-wide**

	**AGRE discovery (208 cases, 151 controls)**						**SSC replication (207 cases, 676 controls)**			
**SNP**	**Allele 1/allele 2**	**Affected allele 1 frequency (%)**	**Unaffected allele 1 frequency (%)**	**Odds ratio**	***P*** **value**	**Corrected** ***P*** **value**	**Affected allele 1 frequency (%)**	**Unaffected allele 1 frequency (%)**	**Odds ratio**	***P*** **value**
Predicted	.	97.0	<24.3	>88	≤1×10^−8^	≤0.05	97.0	<24.3	>88	≤1×10^−8^
rs2387667	G/A	33.7	13.2	2.54	1.1×10^−5^	1.0	15.9	19.5	0.82	0.25
rs867764	A/G	33.7	13.2	2.54	1.1×10^−5^	1.0	23.2	23.5	0.99	0.92
rs10518301	A/G	25.0	47.0	0.53	1.4×10^−5^	1.0	40.1	41.1	0.98	0.79
rs9302760	A/G	75.0	53.0	1.42	1.4×10^−5^	1.0	60.4	56.4	1.07	0.31
rs4745013	G/A	36.1	58.9	0.61	1.7×10^−5^	1.0	49.8	47.8	1.04	0.62

### Exploratory association analyses

Finally, we considered the possibility that our inability to detect the hypothesized single FPE locus was due to inaccurate differentiation of females with, and without, the FPE. For instance, a female may be unaffected due to the absence of risk factors despite absent FPE. We therefore tried defining cases and controls by their SRS score rather than by categorical ASD diagnoses. No SNPs were significant after multiple comparisons (Additional file 1: Figures S5 to S8 and Additional file 1: Table S5). We also considered whether extremes of the affected and unaffected SRS distributions might be enriched for females in whom the FPE was present or absent (Additional file 1: Figure S11). Again, no SNPs were significant after multiple comparisons (Additional file 1: Table S5). In addition, we performed all of the reported analyses under an additive model; no genome-wide significant SNPs were identified (Additional file 1: Figure S9 and S10, Additional file 1: Table S6 and S7).

## Discussion

The observation of a bimodal SRS distribution in females, but not males, from multiplex families raised the possibility of a single genetic locus mediating a female protective effect and resulting in a 4:1 sex bias in ASD. Given the potential of such a locus as a therapeutic target, and the high likelihood that such a locus would be missed by a GWAS with mixed sexes, we performed an association study in females only, which was well powered to detect such an effect.

We considered three tiers of SNPs based on the *a priori* probability that genomic regions might harbor a single locus for FPE. The first tier considered only SNPs unique to chromosome X that escaped X-inactivation, the second tier considered all SNPs on chromosome X, and the third tier was a full genome-wide association study. No SNPs reached significance after correcting for multiple comparisons in any of the three tiers (Figure 4); furthermore, there was no evidence of replication in the SSC cohort, nor of a SNP in one tier being present in the top five SNPs of the next tier. This result was unchanged by an additive model (Additional file 1: Figure S9 and S10), defining case/control status using the SRS score (Additional file 1: Figures S11, Additional file 1: Table S5), or considering the extremes of the SRS distribution (Additional file 1: Table S5).

The female-only GWAS achieved considerably higher power than a GWAS with both sexes and was extremely well powered to detect a single locus for the FPE even with marked deviation from the expected allele frequency (Additional file 1: Figure S2). We therefore conclude that the FPE is unlikely to be mediated by a single genetic locus. This negative result does not reduce the likelihood of a female protective effect being responsible for the sex bias observed in ASD, nor does it reduce the likelihood of this protection being mediated by a polygenic effect.

There are several explanations for this negative result. First, there may be little variance in the FPE between females. For example, if the FPE was mediated by endogenous estrogen levels above a certain threshold, and all females exceeded this threshold, then the FPE would be constant without genetic or environmental risk factors having an effect. Alternatively, the FPE may vary between females, but this variance is determined by multiple genetic and/or environmental factors, for example, if the extent of FPE was dependent on the degree of endogenous estrogen exposure. Finally, it is possible that a single environmental factor (for example, exogenous estrogen exposure) determines the presence of the FPE, though such a factor would need to act in the majority of females, but not act in the majority of males.

The first explanation (FPE in all females) would not lead to the bimodal SRS distribution that prompted this study (Figure 1H), while the second (multifactorial FPE) could only produce a bimodal distribution if the majority of risk factors targeted a common biological pathway or neurological process (Figure 1F). It is hard to reconcile the third explanation (a single environmental effect) with the consistent sex bias observed across so many studies.

This leads us to consider alternative explanations for the bimodal distribution. We first considered ‘non-biological’ biases in the manner of data collection. One possibility is ascertainment bias, that is, that unaffected males are rare in multiplex families, while unaffected females are detected comparatively frequently. Simulation of multiplex families shows that ascertainment bias and a 4:1 sex bias can induce a bimodal distribution in ASD liability that is more pronounced in females (Additional file 1: Figure S12A and S12B). However, we do not think this is the complete explanation of the SRS distribution since the observed data differs from the expectation of this model in two important respects:

First, the lower distribution in females (Additional file 1: Figure S12B) has a mean over one standard deviation above the general population (equivalent to an SRS score of over 40). However, in the multiplex females (Figure 1B), the mean SRS of the lower distribution females is the same as the general population (SRS of 18).

Second, the simulation required a difference in mean liability between males and females of 0.66 standard deviations (equivalent to an SRS of 12). However, the observed SRS difference between males and females is fourfold lower at 0.17 standard deviations (equivalent to an SRS of 3). If we repeat the simulation using a sex difference of 0.17 standard deviations, we observe little distinction between the male and female distributions (Additional file 1: Figure S12C and S12D).

Therefore, while ascertainment bias may partially explain the bimodal SRS in multiplex females, our analyses suggest that it is not the complete explanation of this phenomenon. Similarly, the effect may be a consequence of the sex of the parent rating the child for the SRS score. However, we note that no such rater bias was detected in epidemiologic sample of twins [24,34] and the bimodal distribution has been observed for SRS scored by both parents and teachers [23]. Finally, we considered whether IQ could confound the SRS score; however, we observed very weak correlation between the two measures with a similar slope in males and females (Additional file 1: Figure S13).

We next considered ‘biological’ explanations for the bimodal distribution. The ‘single locus’ observed may represent multiple rare risk factors rather than a single common protective factor, for example, inherited large copy number variation (CNV). The distribution may also be a consequence of more complex interactions between multiple factors mediating protection and risk. For example, a general population twin study [24] observed that reciprocal social behavior in females, but not males, was influenced by rearing factors that operated in the direction of promoting social competency. Further exploration of the manner in which inherited liability to ASD might capitalize upon, or accentuate, developmental sexual dimorphisms in gene expression, neuroanatomy, or behavior is warranted. We note that a large family study [22] observed that a high proportion of the unaffected sisters of ASD probands manifested histories of early language delay with autistic qualities of speech which later resolved. These observations offer potential clues to the manner in which FPE might offset risk in the setting of autism susceptibility early in life.

Microarray and exome sequencing studies have observed an excess *de novo* mutation burden in ASD affected females compared to ASD affected males [2,17-19]; however, a quantitative relationship was not observed between CNV trait burden and ASD symptom severity. This underscores the possibility that FPE operates in a dichotomous manner, either offering complete protection from ASD risk or being completely overwhelmed by an excess of ASD risk.

## Conclusions

In summary, the distribution of ASD severity in females raised the possibility of an ASD protective effect in females mediated by a single genetic locus. If present, such a locus is likely to have been missed by prior GWAS analyses and would have great potential as a therapeutic target. However, we performed a well-powered targeted association study that found no evidence of such a genetic locus. The FPE remains of great interest as a route to discovering therapeutic targets; however, the mechanism of this protection remains unknown.

## Availability of supporting data

The datasets supporting the results of this article are available in the repositories: Simons Foundation Autism Research Initiative, SFARI [http://sfari.org/sfari-initiatives/simons-simplex-collection] and the National Institutes of Health database of Genotypes and Phenotypes, dbGaP [http://www.ncbi.nlm.nih.gov/gap/].

## Additional files

Additional file 1:
**Supplementary online materials.** The file contains supplementary figures, tables, and methods. Additional file 2:
**Regions of X-inactivation.** The file contains a list of chromosome coordinates describing the regions on chromosome X that were identified as escaping X-inactivation. Additional file 3:
**Sample names.** The file contains a list of AGRE and SSC samples included in the analysis.

## Abbreviations

AGRE: Autism Genetic Resource Exchange
ASD: autism spectrum disorder
CNV: copy number variation
FPE: female protective effect
GWAS: genome-wide association study
IAN: Interactive Autism Network
SNP: single nucleotide polymorphism
SRS: Social Responsiveness Scale
SSC: Simons Simplex Collection
