# Supplementary material for: The female protective effect in autism spectrum disorder is not mediated by a single genetic locus
Source: Mol Autism. 2015 May 13;6:25. doi: 10.1186/s13229-015-0014-3 (PMC4429476; doi:10.1186/s13229-015-0014-3)
Supplement: Additional file 1: — Supplementary online materials. The file contains supplementary figures, tables, and methods. [file 13229_2015_14_MOESM1_ESM.pdf]

List of Figures

S1 **A single locus hypothesis for the female protective effect.** 2
S2 **Power to detect a single locus FPE** 3
S3 **Ancestry Analysis.** 4
S4 **Identity by Descent (IBD) Analysis.** 5
S5 **Manhattan plot of association results under a dominant model in the AGRE cohort.** 6
S6 **Manhattan plot of association results under a dominant model in the SSC cohort.** 7
S7 **Manhattan plot of association results under a recessive model in the AGRE cohort.** 8
S8 **Manhattan plot of association results under a recessive model in the SSC cohort.** 9
S9 **Manhattan plot of association results under an additive model in the AGRE cohort.** 10
S10 **Manhattan plot of association results under an additive model in the SSC cohort.** 11
S11 **Stratification of AGRE affected and unaffected females by SRS score.** 12
S12 **Effect of ascertainment bias on ASD liability** 13
S13 **IQ Distribution in SSC Probands** 14
S14 **Genotyping Cluster Plots of all SNPs in Table 1** 15
S15 **Genotyping Cluster Plots of all SNPs in Table 2** 16
S16 **Genotyping Cluster Plots of all SNPs in Table 3** 17
S17 **Genotyping Cluster Plots of SNPs in Exploratory Analysis Table S5** 23

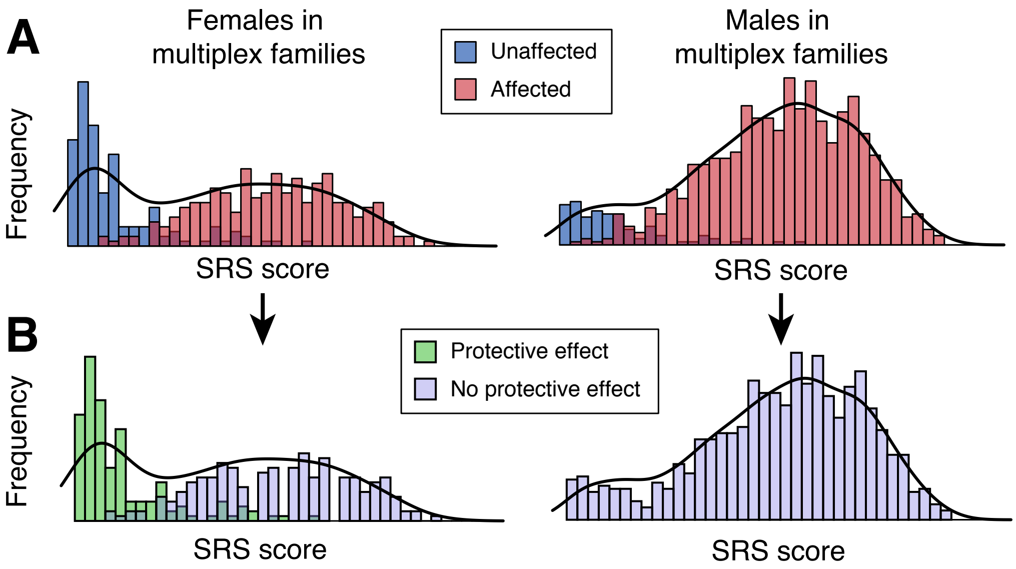

Figure S1. A single locus hypothesis for the female protective effect.
A) A bimodal distribution of ASD risk, measured with the SRS, is observed for females from multiplex families but is less distinct in males. **B)** This bimodal distribution may reflect females with a protective effect (in green) vs. females without such a protective effect (in purple).

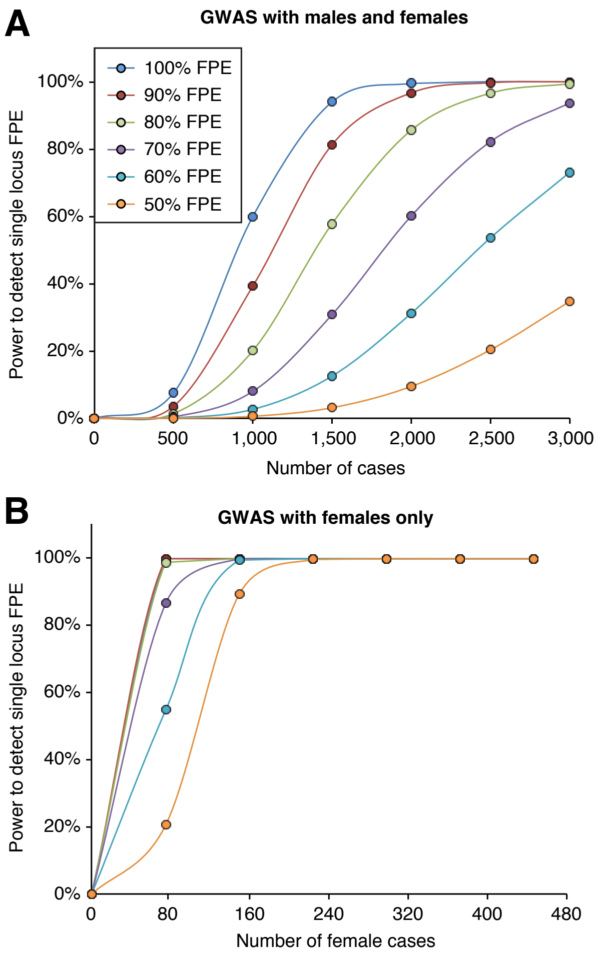

Figure S2. Power to detect a single locus FPE

A) Power was estimated based of the predicated allele frequencies (Table S2) in affected females vs. population females. To model deviation from ideal conditions, the contribution of the FPE to ASD sex bias was decreased from 100% to 50%. For a given number of cases, and an equivalent number of controls, the estimated power is shown. For comparison, the largest GWAS in ASD used 2,678 cases and pseudo-controls Anney et al. (2012). **B)** The analysis is repeated for females only, based on the observed rate of 16% Anney et al. (2012). The power is consistently greater in this female only analysis than in a conventional GWAS.

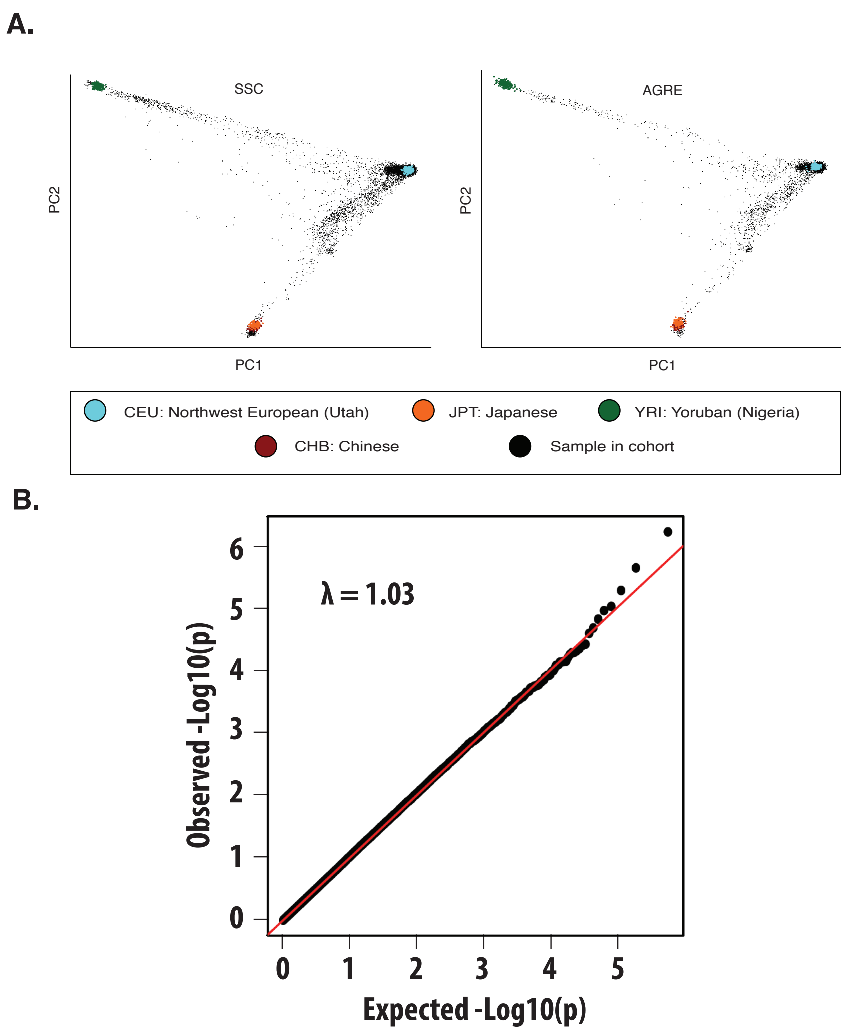

Figure S3. Ancestry Analysis.

A) Population stratification was preformed in EIGENSTRAT. HapMap samples CEU (blue), YRI (green), JPT (Orange), and CHB (red) were used to stratify the AGRE and SSC cohort. **B)** QQ-Plot analysis preformed on a GWAS of AGRE samples yielded a genomic inflation (λ) of 1.03.

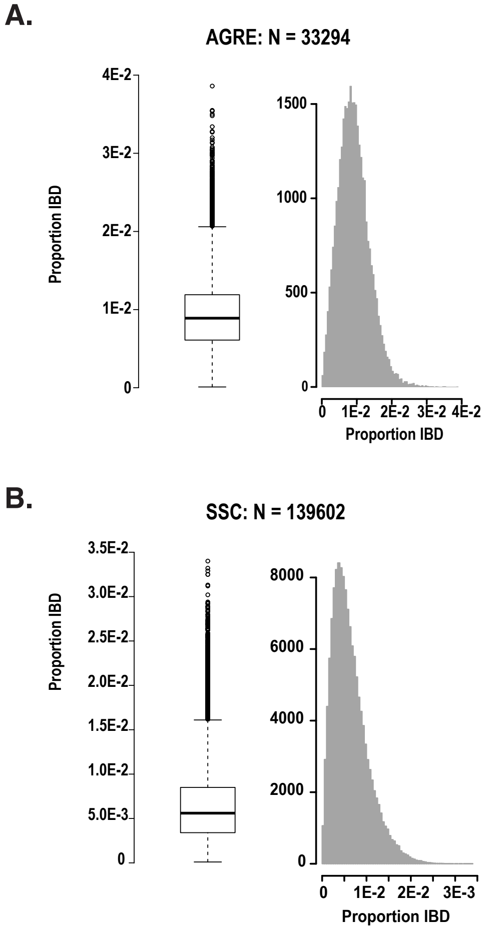

Figure S4. Identity by Descent (IBD) Analysis.

A) 33,294 (51.8%) of the pairwise comparisons between samples in the AGRE cohort registered an IBD proportion greater than 0. All pairwise IBD comparisons yielded a value below 4%. **B)** 139,602 (35.9%) of the pairwise comparisons between samples in the SSC cohort registered an IBD proportion greater than 0. All pairwise IBD comparisons yielded a value below 3.5%.

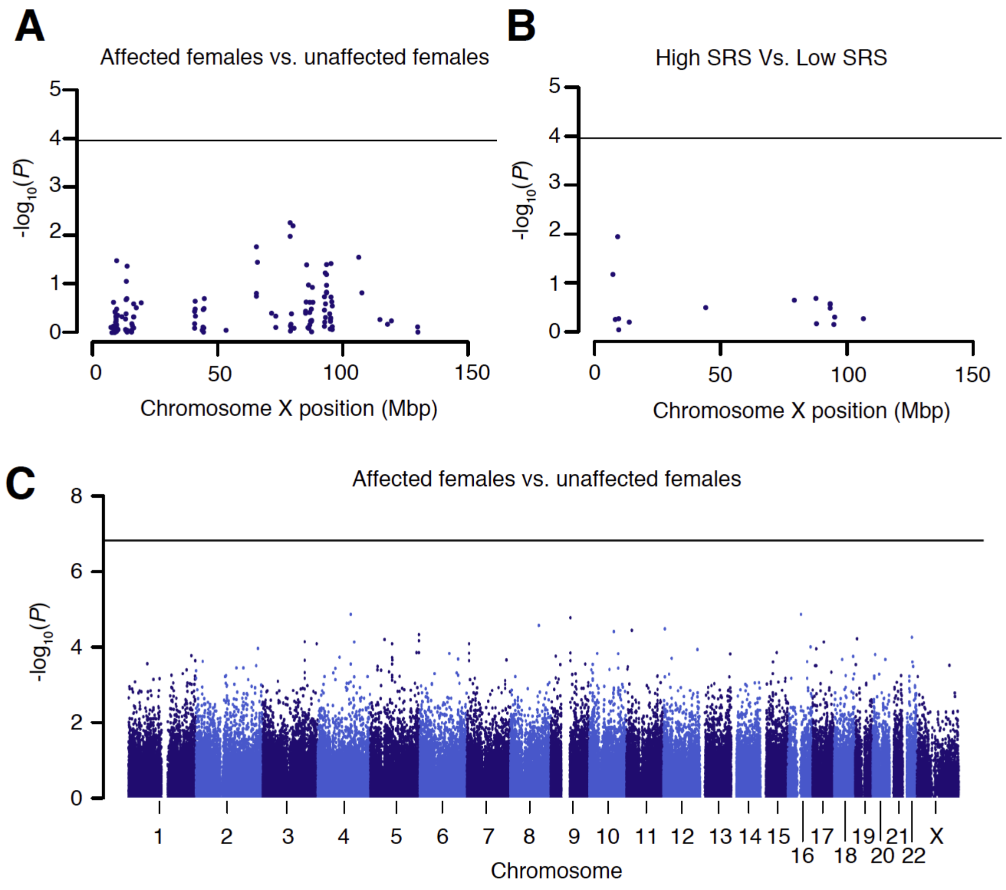

Figure S5. Manhattan plot of association results under a dominant model in the AGRE cohort.

A) Loci on the X-Chromosome within regions shown to escape X-inactivation were examined between cases and controls in AGRE. The association test results under a dominant model are shown. **B)** The analysis was repeated using an SRS cut off value of 45 was used to distinguish cases (high SRS) from controls (low SRS), instead of ASD diagnostic status. **C)** Using ASD diagnosis to identify cases and controls a genome-wide association analysis with a dominant model was performed.

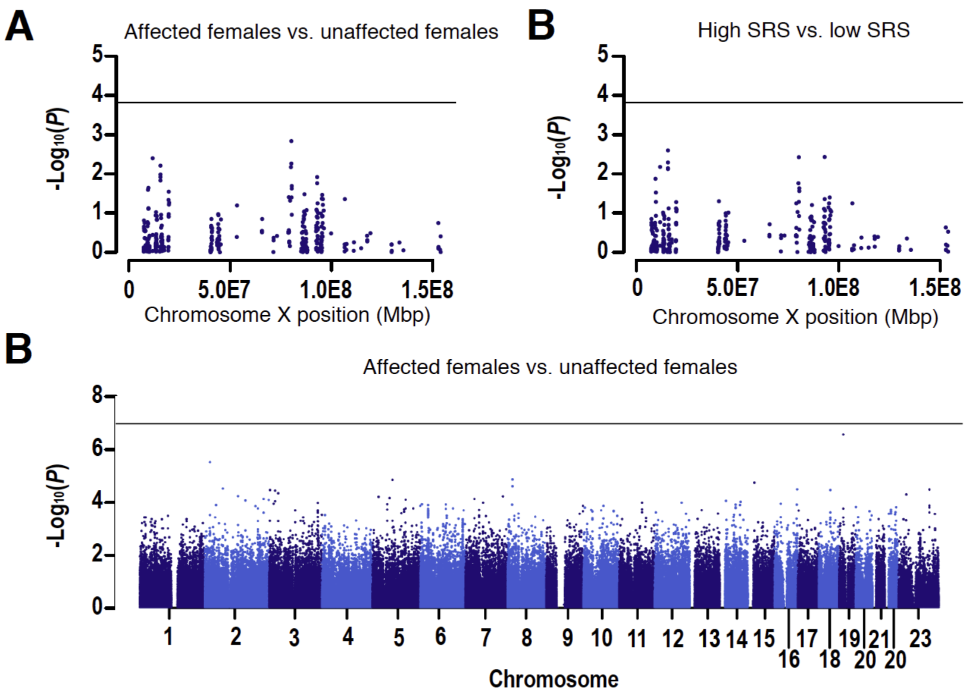

Figure S6. Manhattan plot of association results under a dominant model in the SSC cohort.

A) Loci on the X-Chromosome within regions shown to escape X-inactivation were examined between cases and controls in SCC. The association test results under a dominant model are shown. **B)** The analysis was repeated using an SRS cut off value of 45 was used to distinguish cases (high SRS) from controls (low SRS), instead of ASD diagnostic status. **C)** Using ASD diagnosis to identify cases and controls a genome-wide association analysis with a dominant model was performed.

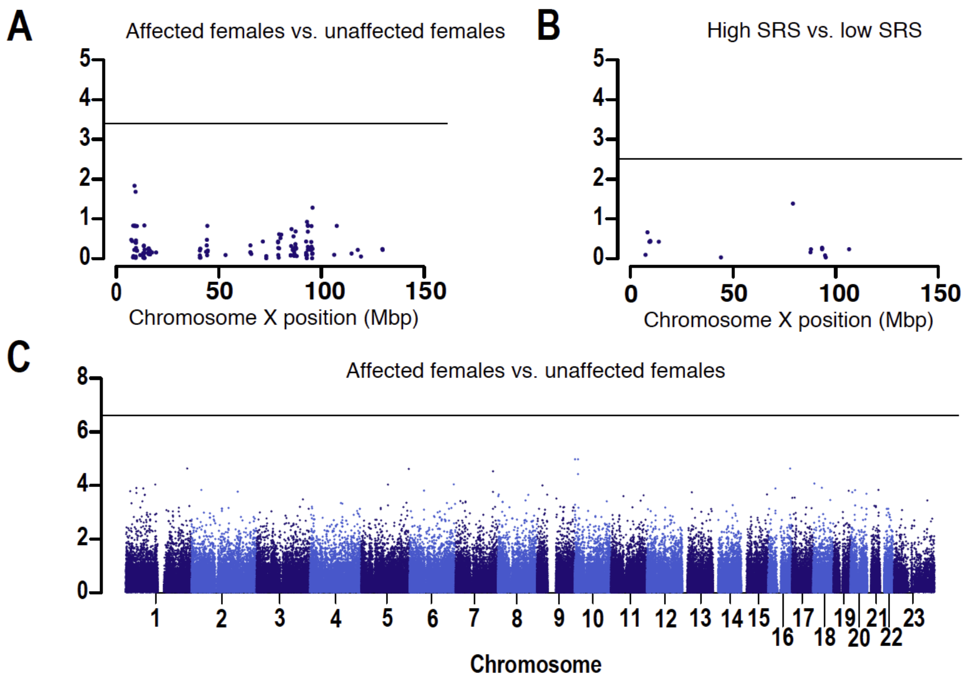

Figure S7. Manhattan plot of association results under a recessive model in the AGRE cohort.

A) Loci on the X-Chromosome within regions shown to escape X-inactivation were examined between cases and controls in AGRE. The association test results under a recessive model are shown. **B)** The analysis was repeated using an SRS cut off value of 45 was used to distinguish cases (high SRS) from controls (low SRS), instead of ASD diagnostic status. **C)** Using ASD diagnosis to identify cases and controls a genome-wide association analysis with a recessive model was performed.

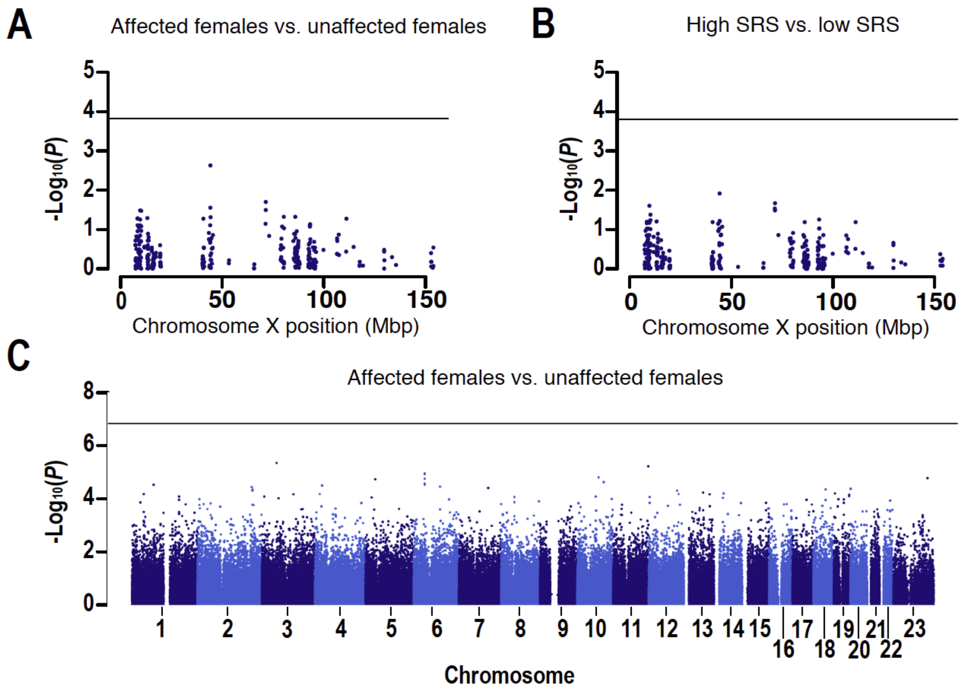

Figure S8. Manhattan plot of association results under a recessive model in the SSC cohort.

A) Loci on the X-Chromosome within regions shown to escape X-inactivation were examined between cases and controls in SSC. The association test results under a recessive model are shown. **B)** The analysis was repeated using an SRS cut off value of 45 was used to distinguish cases (high SRS) from controls (low SRS), instead of ASD diagnostic status. **C)** Using ASD diagnosis to identify cases and controls a genome-wide association analysis with a recessive model was performed.

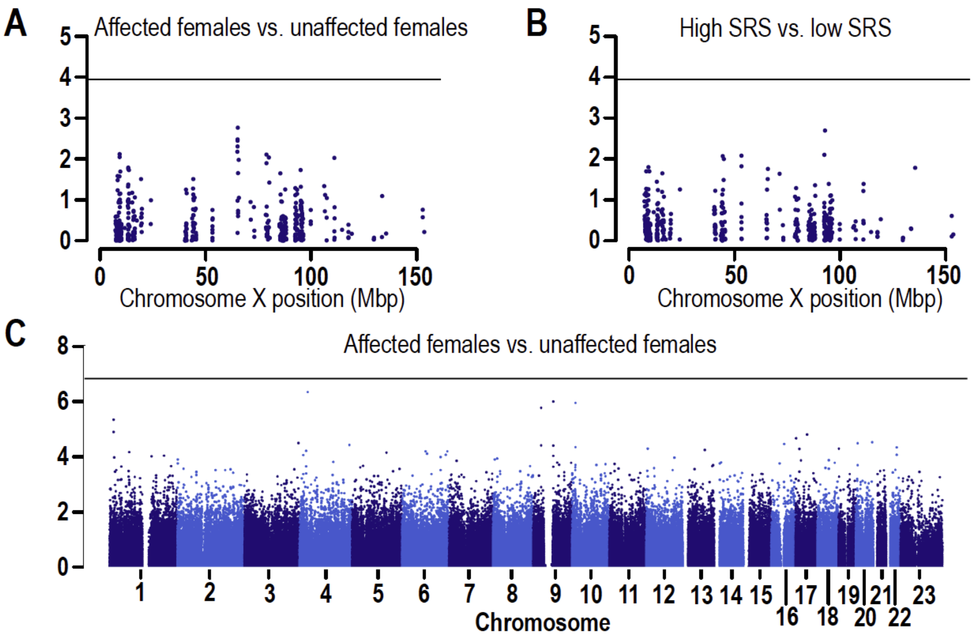

Figure S9. Manhattan plot of association results under an additive model in the AGRE cohort.

A) Loci on the X-Chromosome within regions shown to escape X-inactivation were examined between cases and controls in SSC. The association test results under a recessive model are shown. **B)** The analysis was repeated using an SRS cut off value of 45 was used to distinguish cases (high SRS) from controls (low SRS), instead of ASD diagnostic status. **C)** Using ASD diagnosis to identify cases and controls a genome-wide association analysis with a recessive model was performed.

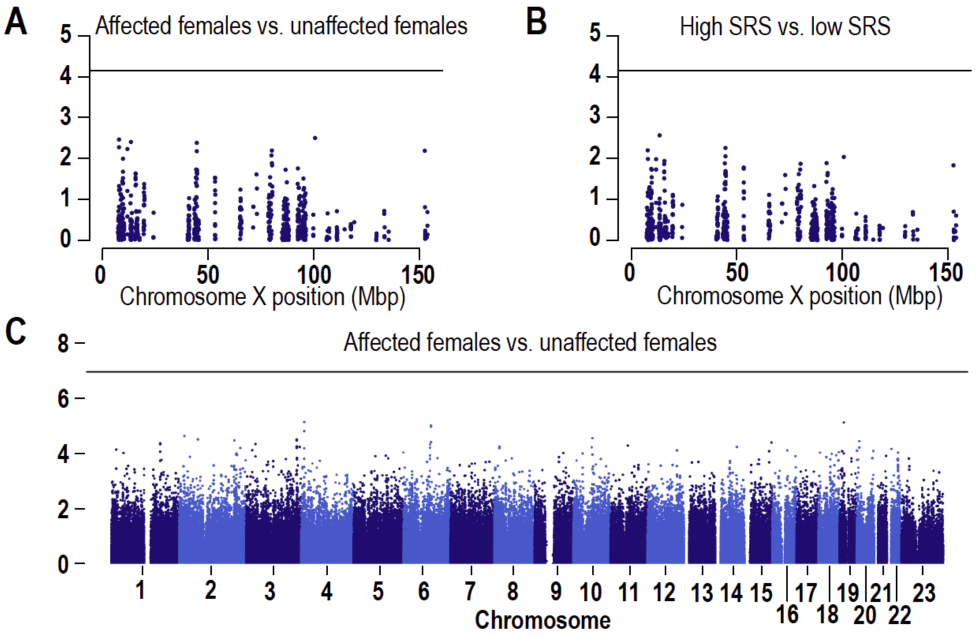

Figure S10. Manhattan plot of association results under an additive model in the SSC cohort.

A) Loci on the X-Chromosome within regions shown to escape X-inactivation were examined between cases and controls in SSC. The association test results under a recessive model are shown. **B)** The analysis was repeated using an SRS cut off value of 45 was used to distinguish cases (high SRS) from controls (low SRS), instead of ASD diagnostic status. **C)** Using ASD diagnosis to identify cases and controls a genome-wide association analysis with a recessive model was performed.

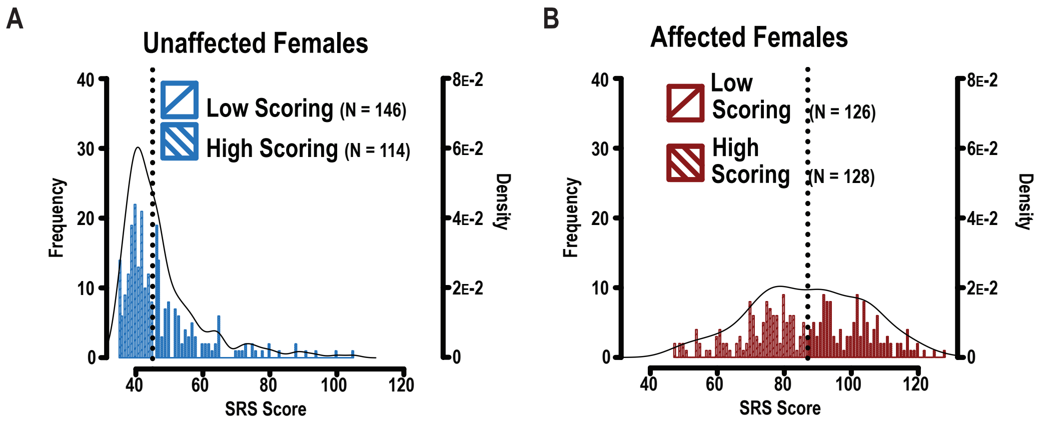

Figure S11. Stratification of AGRE affected and unaffected females by SRS score. **A)** Unaffected females were stratified into a higher scoring (N=114) and lower scoring (N=146) population based on an SRS score of 45. After data cleaning, and considering only unrelated samples of European ancestry, the samples sizes were 54 and 64 respectively. **B)** Affected females were stratified at the 50th percentile into higher scoring (N=128) and lower scoring (N=126) groups. After data cleaning, and considering only unrelated samples of European ancestry, the samples sizes were 58 and 51 respectively.

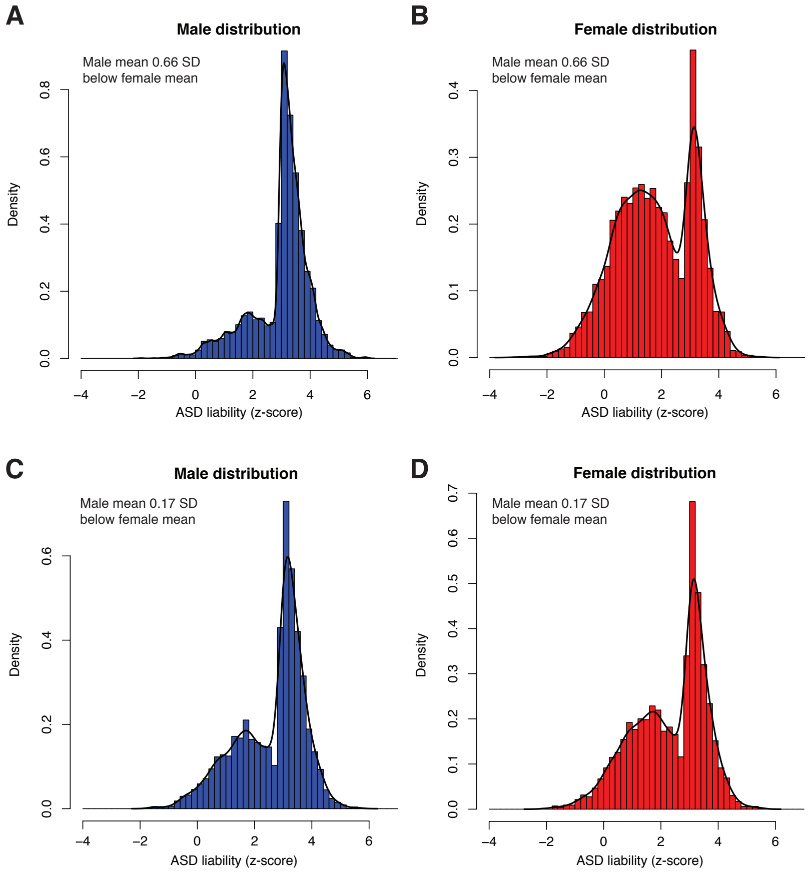

Figure S12. Effect of ascertainment bias on ASD liability

A) Distribution of ASD liability in males in simulated families selected using the ascertainment methods used in the AGRE collection (at least two affected children). The male and female mean liability was shifted by 0.66 standard deviations to give a 4:1 sex bias and the diagnostic threshold was selected to give an incidence of 1%. **B)** The female liability distribution under the same model as 'A'. **C)** The simulation was repeated using a shift of 0.17 standard deviations which is equivalent to the difference of 3 SRS points observed between male and female samples. **D)** The female liability distribution under the same model as 'C'.

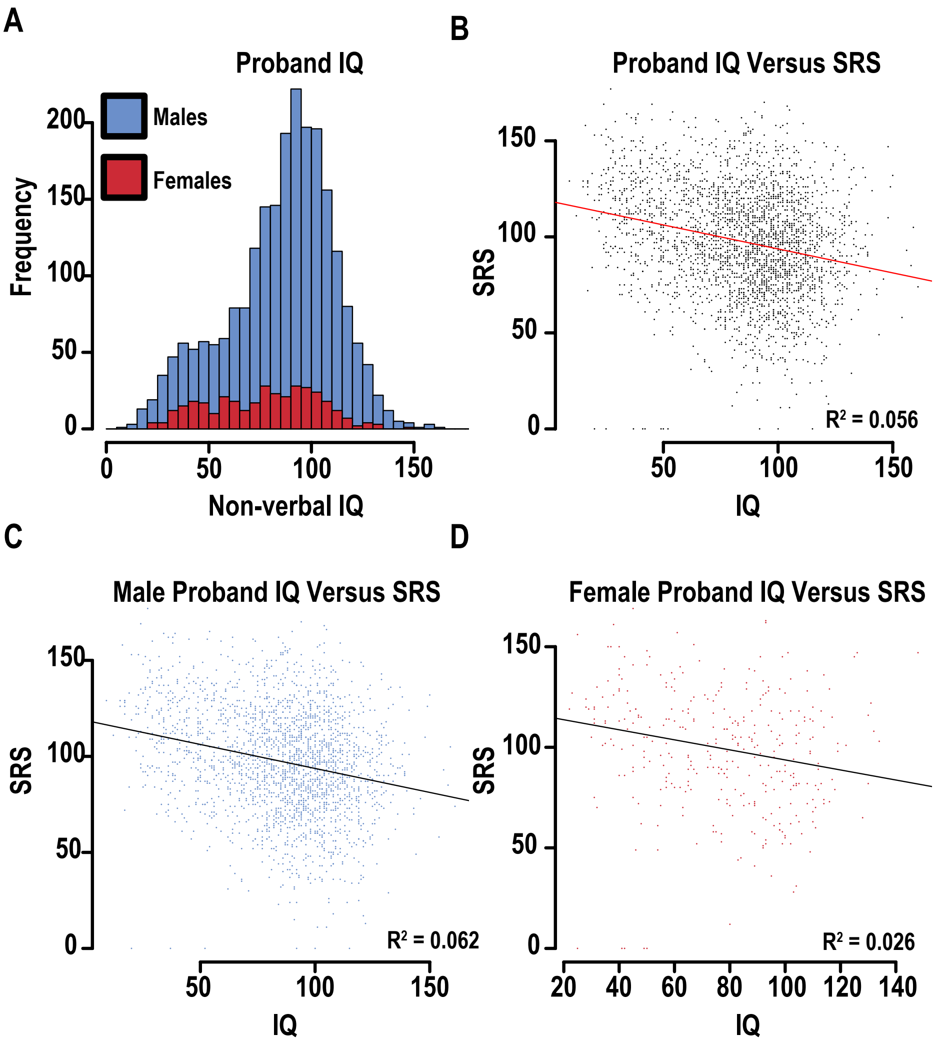

Figure S13. IQ Distribution in SSC Probands

A) Histogram of male (blue) and Female (red) IQ distributions in SSC probands. **B)** Plot of SRS as a function of IQ in SSC probands. Linear regression model was used to generate the best fit line (red) and correlation value (R^2). **C)** Plot of SRS as a function of IQ in male SSC probands. Linear regression model was used to generate the best fit line (red) and correlation value (R^2). **D)** Plot of SRS as a function of IQ in female SSC probands. Linear regression model was used to generate the best fit line (red) and correlation value (R^2).

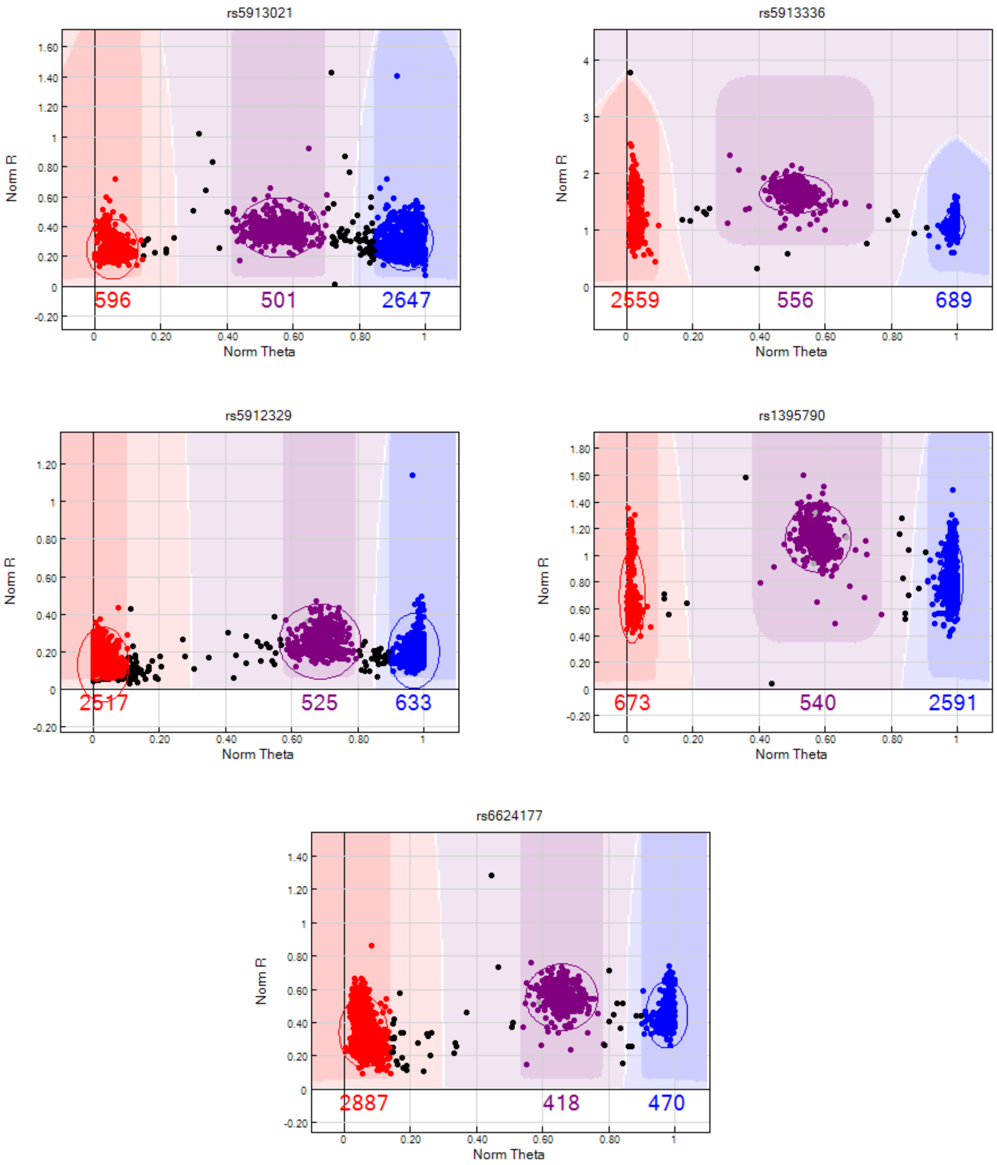

Figure S14. Genotyping Cluster Plots of all SNPs in Table 1

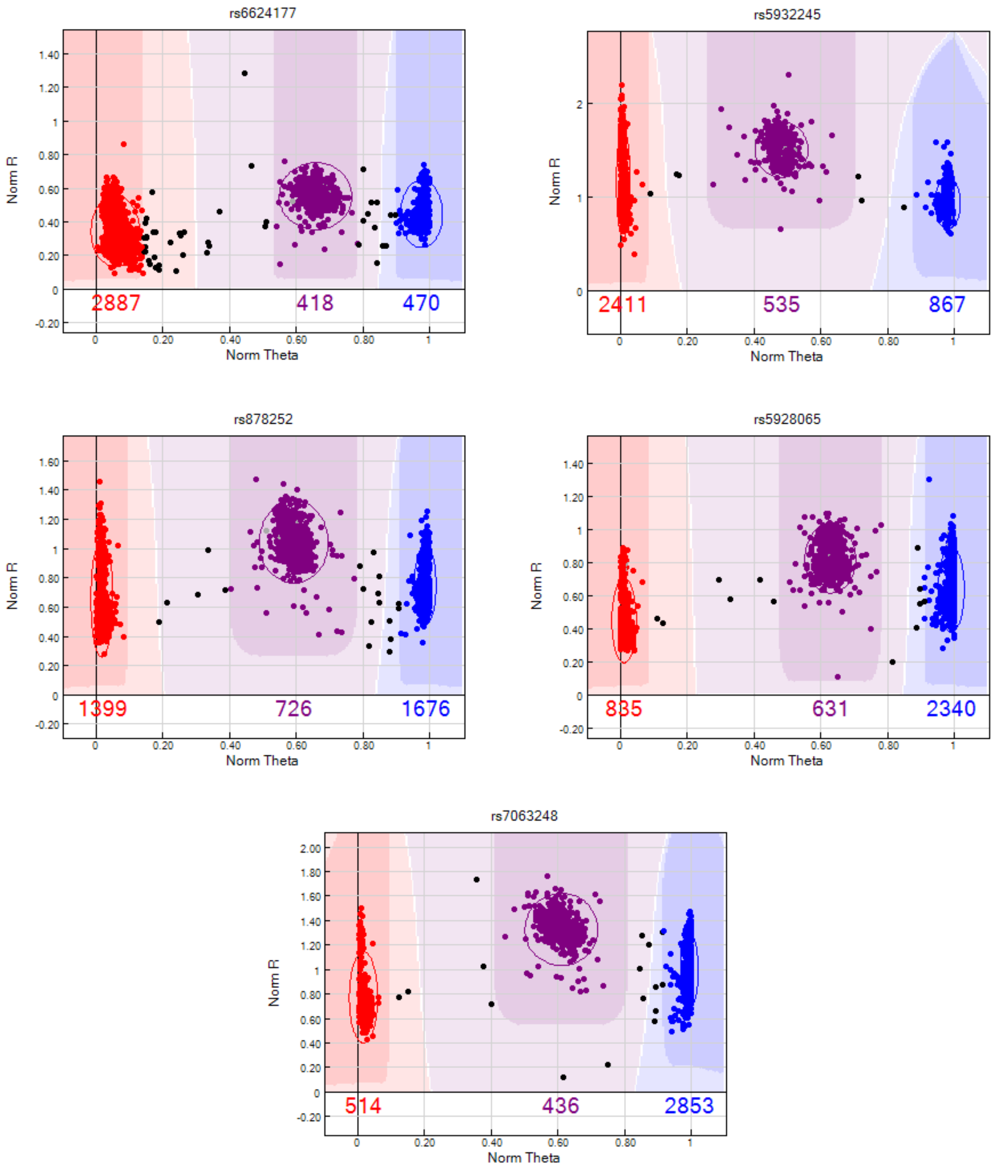

Figure S15. Genotyping Cluster Plots of all SNPs in Table 2

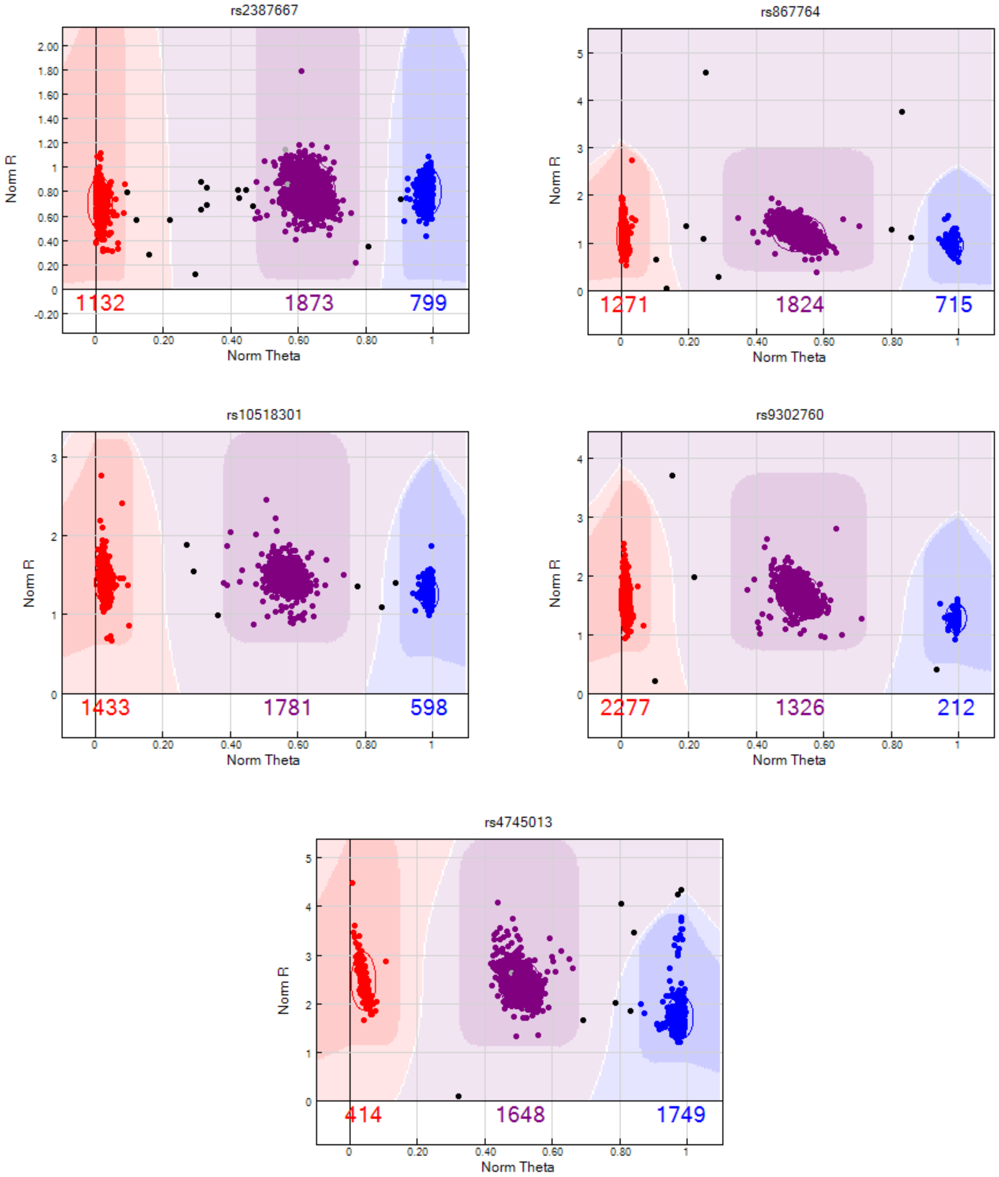

Figure S16. Genotyping Cluster Plots of all SNPs in Table 3

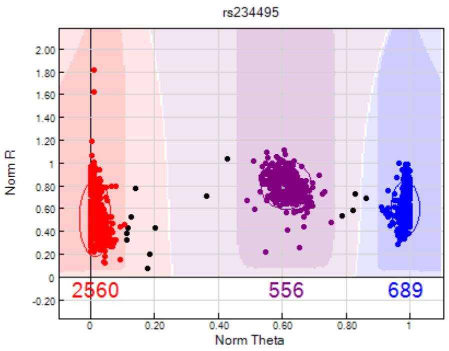
rs234495
Norm R
Norm Theta
2560
556
689

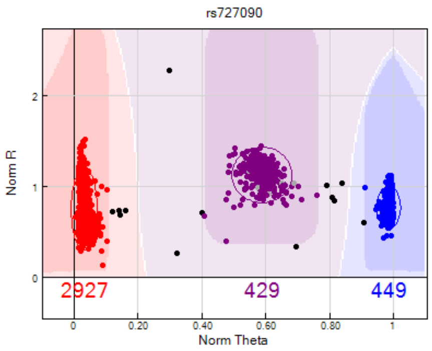
rs727090
Norm R
Norm Theta
2927
429
449

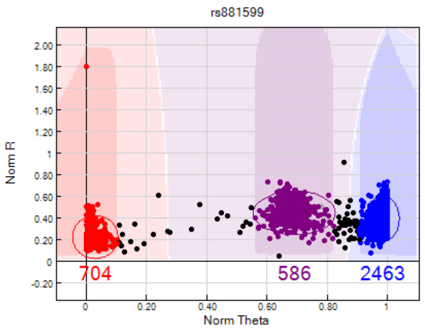
rs881599
Norm R
Norm Theta
704
586
2463

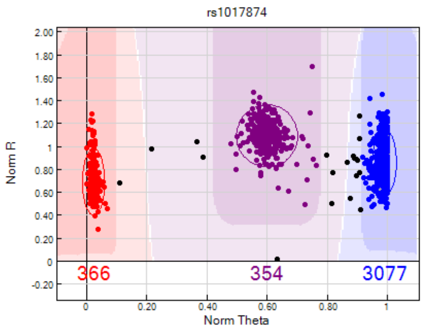
rs1017874
Norm R
Norm Theta
366
354
3077

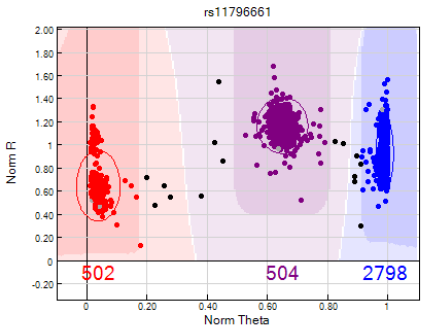
rs11796661
Norm R
Norm Theta
502
504
2798

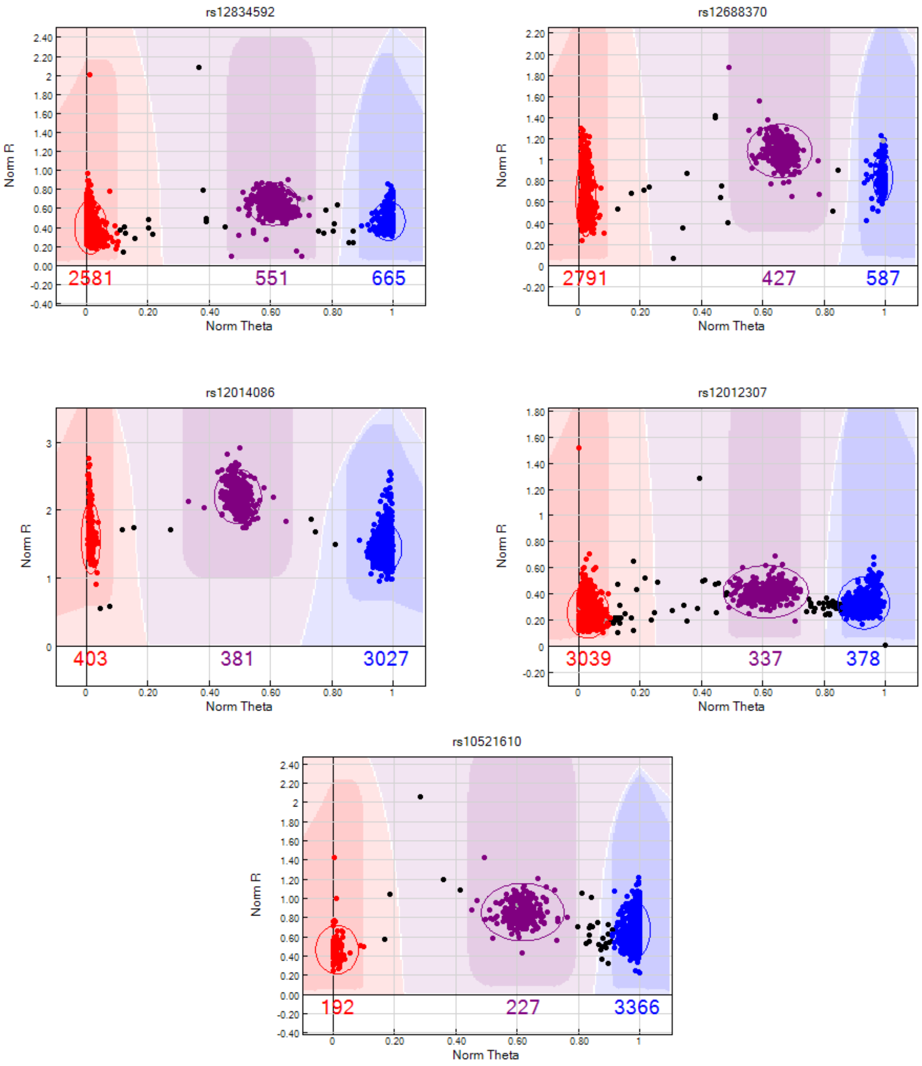
rs12834592
Norm R
Norm Theta
2581
551
665
rs12688370
Norm R
Norm Theta
2791
427
587
rs12014086
Norm R
Norm Theta
403
381
3027
rs12012307
Norm R
Norm Theta
3039
337
378
rs10521610
Norm R
Norm Theta
192
227
3366

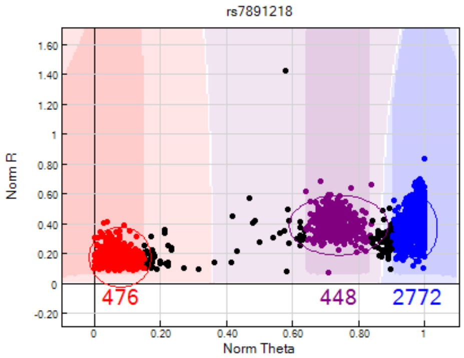
rs7891218
Norm R
Norm Theta
476
448
2772

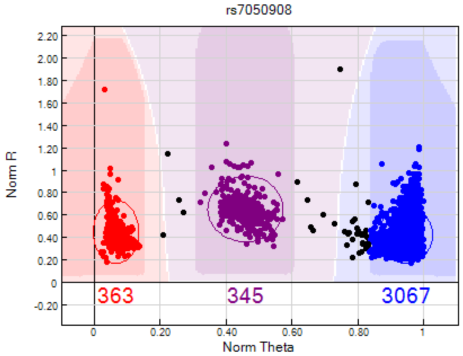
rs7050908
Norm R
Norm Theta
363
345
3067

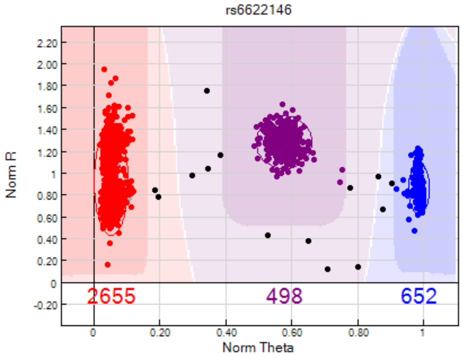
rs6622146
Norm R
Norm Theta
2655
498
652

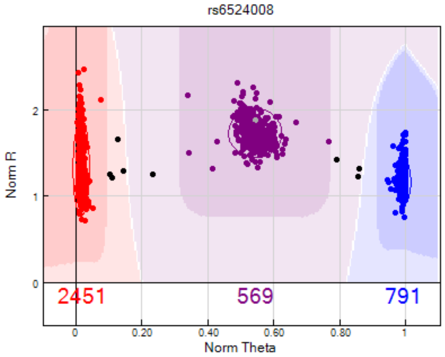
rs6524008
Norm R
Norm Theta
2451
569
791

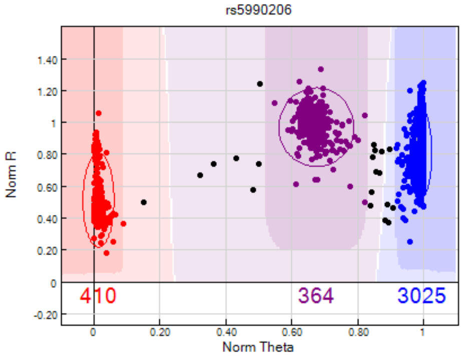
rs5990206
Norm R
Norm Theta
410
364
3025

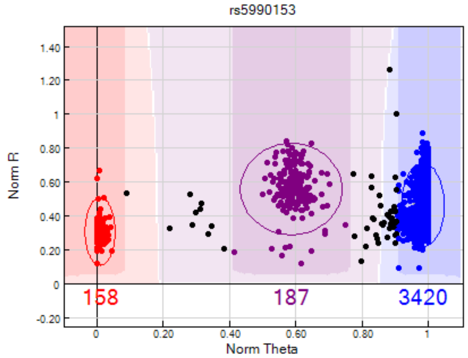
rs5990153
Norm R
Norm Theta
158
187
3420

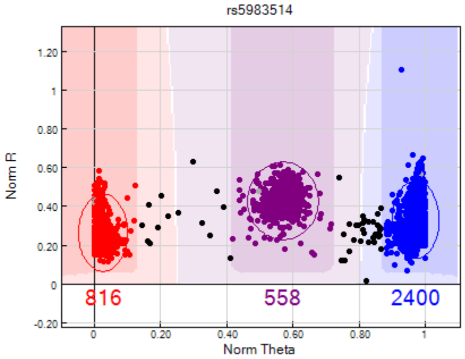
rs5983514
Norm R
Norm Theta
816
558
2400

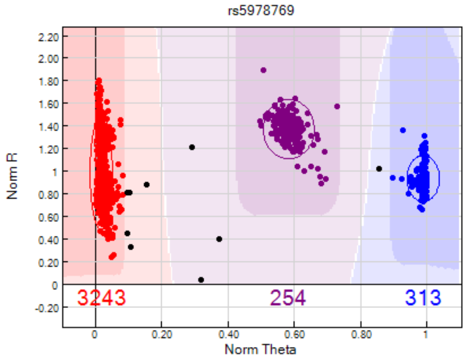
rs5978769
Norm R
Norm Theta
3243
254
313

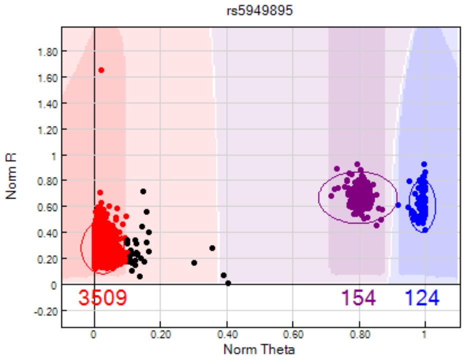
rs5949895
Norm R
Norm Theta
3509
154
124

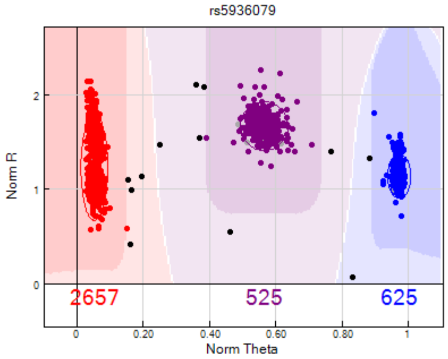
rs5936079
Norm R
Norm Theta
2657
525
625

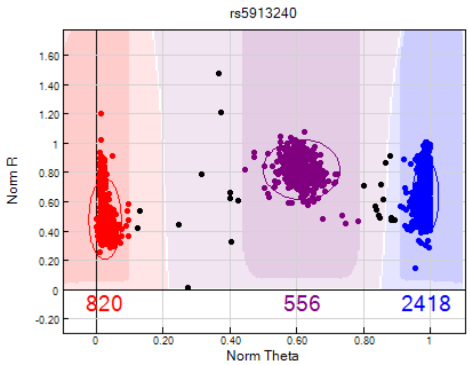
rs5913240
Norm R
820
556
2418
Norm Theta

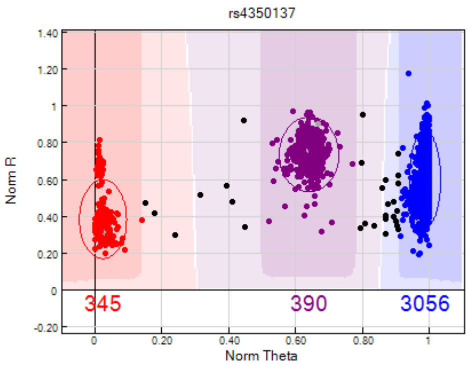
rs4350137
Norm R
345
390
3056
Norm Theta

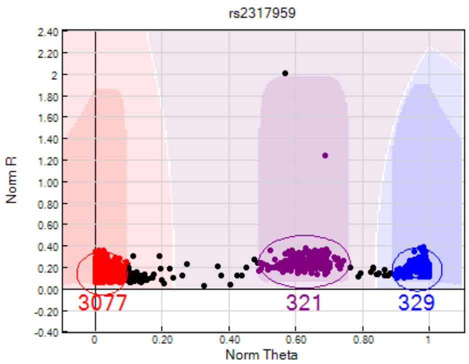
rs2317959
Norm R
3077
321
329
Norm Theta

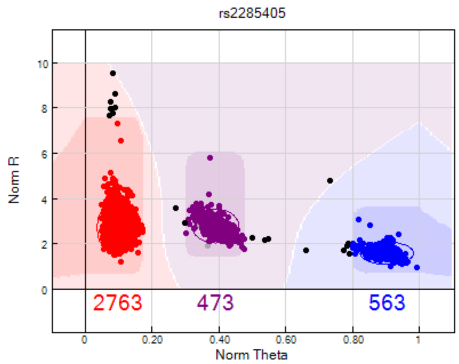
rs2285405
Norm R
2763
473
563
Norm Theta

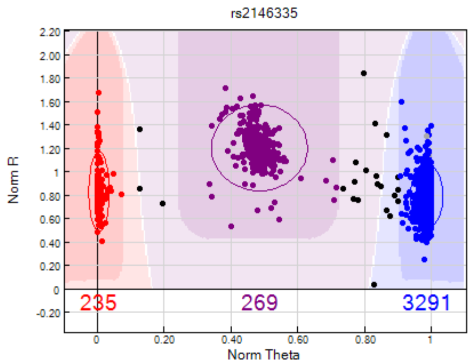
rs2146335
Norm R
235
269
3291
Norm Theta

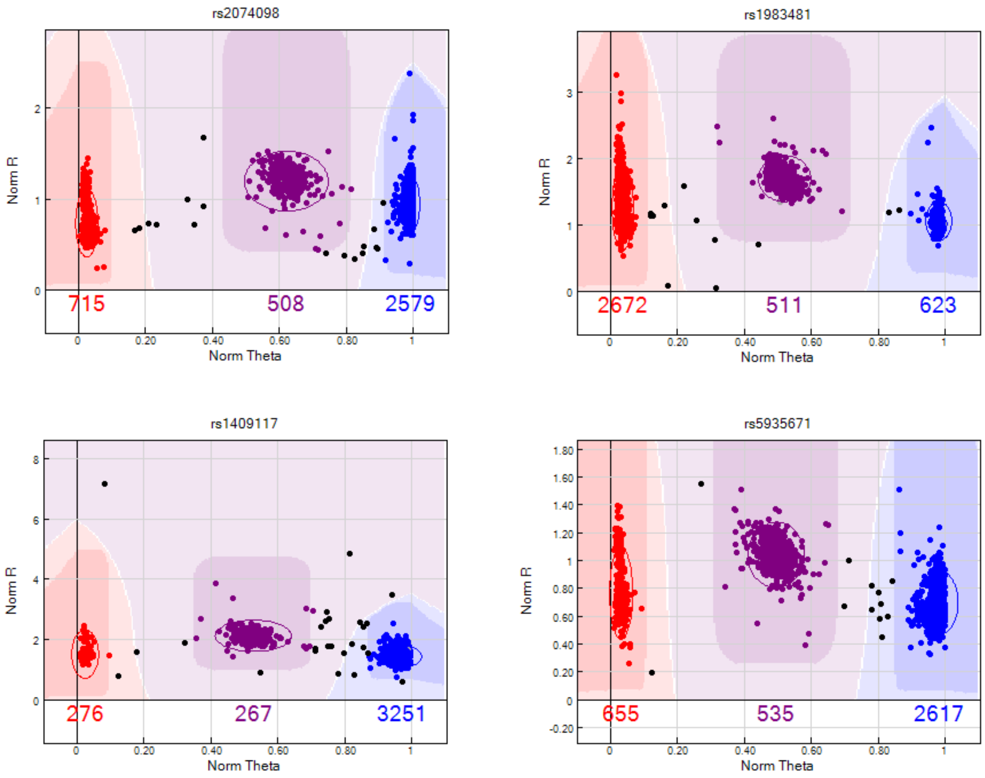

Figure S17. Genotyping Cluster Plots of SNPs in Exploratory Analysis Table S5

List of Tables

S1 **Predicted allele frequency of single locus FPE** 25
S2 **Predicted frequency of p risk allele under a dominant model in cases and controls** 26
S3 **FPE Samples (.xlsx file)** 27
S4 **Regions that escape X-inactivation (.xlsx file)** 28
S5 **AGRE Association test results based on SRS defined case/-control definitions** 29
S6 **Top SNPs from Additive Model analysis of the AGRE cohort** 30
S7 **Top SNPs from Additive Model analysis of the SSC cohort** 31

Category	PP	Pp	pP	pp	P	p
Sex	Female				Male	
Protective effect	Present	Absent			Absent	
Population Frequency	37.8%	5.7%	5.7%	0.8%	43.5%	6.5%
ASD incidence	0.016%	1.6%	1.6%	1.6%	1.6%	1.6%
Population ASD incidence	0.006%	0.09%	0.09%	0.01%	0.70%	0.10%
Population ASD incidence	0.20%				0.80%	
Sex ratio	1.0				4.0	
Total population incidence	1.00%					

Table S1. Predicted allele frequency of single locus FPE

The presence of a simple bimodal distribution raises the possibility that a protective allele (P) at a single locus could produce the female protective effect; protection mediated by two copies of the allele (PP) would be limited to females. Furthermore, a population allele frequency of 87% for P, and 13% for the non-protective p allele, leads to the observed ASD sex ratio of 4:1. "Population frequency" is calculated by: 50% (percent of population of that sex) x allele 1 frequency x allele 2 frequency. "ASD incidence" is estimated based on a total ASD incidence of 1.0% and an arbitrary 100x reduction in incidence when the female protective effect is present. "Population ASD incidence" is estimated by "Population frequency" multiplied by "ASD incidence" and these are summed to give "ASD incidence" by sex and "Total population incidence". The ratio of "ASD incidence" by sex gives the "Sex ratio". A P allele frequency of 87% is the only value to produce a 4:1 sex ratio and 1% ASD incidence. Decreasing the effectiveness of the FPE from 100-fold requires the allele frequency of P to increase.

Category	PP	Pp	pP	pp
Frequency in females	75.69%	11.31%	11.31%	1.69%
Frequency in affected females	3.02%	45.12%	45.12%	6.74%
Frequency in unaffected females	99.68%	0.15%	0.15%	0.02%

Table S2. Predicted frequency of p risk allele under a dominant model in cases and controls

The "Frequency in females" of each genotype is estimated from a *P* allele frequency of 87% and a *p* allele frequency of 13%. Under a model where the FPE reduces the incidence of ASD by 100-fold, the *PP* genotype is markedly reduced in affected females. Conversely, if all females have been exposed to risk, as expected in a multiplex family, the unaffected females should be depleted for non-protective genotypes. For power calculations (Figure S2) the more conservative estimate of "Frequency in females" was used to estimate allele frequency in unaffected females, rather than the "Frequency in unaffected females".

Table S3. FPE Samples (.xlsx file)

This excel file shows the samples used in four association tests. Each association test is listed on a separate sheet, with the cohort (AGRE or SSC) and test (Diag or SRS) used to name the sheet. On each sheet the first column shows the family ID, the second column shows the sample ID of an affected female (0 is absent), and the third column shows the sample column of an unaffected female (0 if absent). Each family contains only one affected or one unaffected female. Only one individual per-family was considered. In the "Diag" analyses affected females were compared with unaffected females according to ASD diagnosis and this analysis is presented in the main manuscript. The "SRS" analysis compares females with a high SRS (cases) to females with a low SRS (controls) using an SRS cutoff of 45 which is the SRS score that best distinguishes cases from controls based on ASD diagnosis. The "Diag_SRS" analysis uses a combination of categorical diagnosis and SRS to define cases and controls in AGRE only.

Table S4. Regions that escape X-inactivation (.xlsx file)

This excel file lists the regions determined to escape X-inactivation. The columns show chromosome, start, stop (BED format) in the hg18 genome build.

Comparison	SNP	Chr	Position	P-Val	BF-Corrected
High SRS Versus Low SRS	rs7891218	X	92838948	2.0E-03	0.91
	rs5983514	X	92560942	7.9E-03	1
	rs1409117	X	53319220	8.4E-03	1
	rs2146335	X	44389803	8.6E-03	1
	rs7050908	X	44918083	1.0E-02	1
Affected, high SRS Versus Affected, low SRS	rs6524008	X	107483616	4.4E-03	1
	rs6622146	X	106209321	4.6E-03	1
	rs12014086	X	44065595	6.9E-03	1
	rs5990206	X	95558656	9.6E-03	1
	rs10521610	X	10124322	1.0E-02	1
Affected, high SRS Versus Unaffected, high SRS	rs12012307	X	19803729	3.4E-03	1
	rs4350137	X	93943526	4.2E-03	1
	rs727090	X	13469667	4.6E-03	1
	rs1017874	X	19779567	5.7E-03	1
	rs2285405	X	119262477	8.7E-03	1
Affected, high SRS Versus Unaffected, low SRS	rs881599	X	9135603	2.7E-03	1
	rs2317959	X	94641848	3.9E-03	1
	rs2074098	X	13735538	5.3E-03	1
	rs5978769	X	7808157	1.5E-02	1
	rs12688370	X	13806166	1.6E-02	1
Affected, low SRS Versus Unaffected, high SRS	rs5935671	X	13786582	2.0E-03	0.89
	rs5990206	X	95558656	2.9E-03	1
	rs5936079	X	15867223	3.7E-03	1
	rs234495	X	15348936	7.1E-03	1
	rs11796661	X	86749681	8.7E-03	1
Affected, low SRS Versus Unaffected, low SRS	rs6622146	X	106209321	3.1E-03	1
	rs5949895	X	95725873	5.6E-03	1
	rs1983481	X	8292613	7.0E-03	1
	rs5990153	X	95028102	1.3E-02	1
	rs12688370	X	13806166	1.3E-02	1
Unaffected, high SRS Versus Unaffected, low SRS	rs5949895	X	95725873	5.6E-04	0.25
	rs234495	X	15348936	2.9E-03	1
	rs5936079	X	15867223	1.0E-02	1
	rs5913240	X	79384683	1.0E-02	1
	rs12834592	X	92669150	1.2E-02	1

Table S5. AGRE Association test results based on SRS defined case/control definitions

Considering the possibility that the protective status of the females may not correlate well with ASD diagnosis, we compared females in AGRE based on a combination of SRS and ASD categorical diagnosis (Figure S11). The top five SNPs in each analysis are shown.

Comparison	SNP	Chr	Position	P-Val	BF-Corrected
Affected Versus Unaffected	rs1028348	X	65384163	1.7E-03	0.77
	rs670546	X	65272116	3.3E-03	1
	rs5965083	X	65230426	3.6E-03	1
	rs5964488	X	65253769	4.9E-03	1
	rs6525038	X	65193018	6.7E-03	1
High SRS Versus Low SRS	rs7891218	X	92952292	2.0E-03	0.91
	rs5983514	X	92674286	7.9E-03	1
	rs1409117	X	53302495	8.4E-03	1
	rs2146335	X	44504859	8.6E-03	1
	rs7050908	X	45033139	1.0E-04	1
GWAS	rs7668302	4	29852055	4.8E-07	0.15
	rs4745013	9	72277423	1.1E-06	0.34
	rs867764	10	12794868	1.2E-06	0.38
	rs7020846	9	28393443	1.8E-06	0.56
	rs9943244	1	15268468	4.8E-06	1

Table S6. Top SNPs from Additive Model analysis of the AGRE cohort

Comparison	SNP	Chr	Position	P-Val	BF-Corrected
Affected Versus Unaffected	rs6621080	X	100630202	3.2E-03	1
	rs845127	X	7785325	3.4E-03	1
	rs5979883	X	13390855	4.0E-03	1
	rs7058967	X	44592534	4.1E-03	1
	rs16984793	X	7815994	5.3E-03	1
High SRS Versus Low SRS	rs5979883	X	13390855	2.7E-03	0.91
	rs7058967	X	44592534	5.5E-03	1
	rs845127	X	7785325	6.3E-03	1
	rs12846943	X	44607553	8.9E-03	1
	rs6621080	X	100630202	9.2E-03	1
GWAS	rs1454870	4	11884740	7.3E-06	1
	rs12462380	19	16298048	7.7E-06	1
	rs1054227	6	100097577	1.0E-05	1
	rs2296154	6	100099989	1.1E-05	1
	rs2124141	4	11851124	1.6E-06	1

Table S7. Top SNPs from Additive Model analysis of the SSC cohort

Supplemental Experimental Procedures

Contents

1 Hypothesized allele frequency **33**
1.1 Rate of protective *PP* genotype in cases and controls 33

2 Estimating power **33**
2.1 Estimating power under suboptimal conditions 34

3 Defining European ancestry **35**

4 Defining SNPs that escape X-inactivation **35**

5 SRS based subject groupings of females in multiplex autism families **35**

6 Assessing the impact of ascertainment bias on liability **37**

1. Hypothesized allele frequency

All analyses presented in this paper are based on the hypothesis that a single common allele is responsible for a female protective effect (FPE) that produces a 4:1 sex bias in autism. There are three variables that constrain the allele frequency for such an allele: 1) The incidence of autism in the population, we will use an estimate of 1% Fombonne (2009); 2) The observed sex bias, we will use an estimate of 4:1 male to female Fombonne (2009); and 3) the effect of the FPE on the incidence of ASD. Our hypothesis states that the FPE is unevenly distributed between females, being present in some, but absent in others, however we are unaware of any empirical evidence regarding how effective such protection might be under this hypothesis. Therefore, for the purposes of modeling, we assumed that the FPE was highly effective in protecting against ASD, using an arbitrary estimate of a 100-fold reduction in risk.

For consistency across the different tiers of the analysis, we considered a protective major allele (P) and a non-protective minor allele (p) with p acting in a dominant fashion to disrupt protection. Of note, the power calculations are unchanged if the non-protective allele acted in a recessive fashion, as the predicted difference in allele frequency between cases and controls is identical.

Under a dominant model, PP confers protection, while Pp, pP, and pp do not. To produce a 4:1 sex bias and 1% ASD incidence, the P allele must occur at a population frequency of 87% (see Table S1). Under this hypothesis 76% (87% x 87%) of females are protected, while the remaining 24% lack protection.

1.1. Rate of protective PP genotype in cases and controls

Under a model where the FPE reduces ASD incidence by 100-fold, affected females would be expected to be greatly depleted for the protective PP genotype. Table S2 shows the estimated percentage of females with two copies of the P allele in the affected group and in the unaffected group, assuming all unaffected females were exposed to risk. Since only females are considering in all three tiers of the association analysis, these estimated frequencies will not change regardless of whether chromosome X or autosomes are considered.

2. Estimating power

Having estimated the frequency of the PP genotype in affected females, we considered our ability to detect these alleles in an association study. The estimate of allele frequencies in unaffected females (Table S2) assumes that

these subjects were exposed to risk, however this probably not the case for a subset of females due to *de novo* mutations and rare inherited variants acting in a dominant manner. Therefore, the *PP* genotype frequency in the population (Table S2, "Frequency in females") represents a worst case (no enrichment of unprotected females) while the *PP* genotype frequency in unaffected females (Table S2, "Frequency in unaffected females") is a best case scenario. For the purposes of the power estimation, we used the population *PP* genotype frequency as the more conservative approach.

Power was estimated using G*Power3.1 Faul et al. (2007) using the "exact" test family, "Proportions: Inequality, two independent groups (Fisher's exact test)", "Post hoc: Compute achieved power - given α, sample size, and effect size". For power of a GWAS, two tailed analysis was selected, the frequency of the *PP* genotype in affected females was entered as "Proportion p1" and the proportion of the *PP* genotype in unaffected females was entered as "Proportion p2". The value used for "α err prob" was 1e-07. For sample size of each group, the number of cases and controls used was equal (in line with the pseudo-controls used)Anney et al. (2012). The results are shown in Figure S2.

2.1. Estimating power under suboptimal conditions

This model assumes that: 1) The FPE is solely responsible for the 4:1 sex bias; 2) The hypothesized locus is solely responsible for the FPE; 3) ASD diagnosis is 100% accurate in assessing cumulative ASD risk; and 4) The FPE reduces incidence by 100-fold. There is a high likelihood that the population effects differ from one or more of these assumptions; therefore we sort to assess how the power was affected by deviation from this model. To achieve this, we reduced the extent to which the FPE contributed to ASD sex bias. To consider a model where the FPE only contributed 50% of sex bias, the difference between the frequency of the *p* risk allele in affected and unaffected females (97% - 24% = 73%) was halved (73% / 2 = 36.5%) and the new *p* risk allele frequency in affected females was calculated by adding this difference to the *p* risk allele frequency in unaffected females (24% + 36.5% = 60.5%). The power calculation was repeated with this lower estimate of the *p* risk allele in affected females. The results of reducing the FPE contribution are shown in Figure S2.

Reducing the contribution of the FPE to ASD sex bias is a method of testing the relative power of a conventional GWAS versus a sex-specific GWAS for this hypothesis. The decreasing contribution of the FPE can be considered as a proxy for adding noise or general deviation from the optimal predicted conditions. Of

note, the sex-specific GWAS achieves considerably greater power for an equivalent sample size than a conventional GWAS (Figure S2).

3. Defining European ancestry

Ancestry was examined as described in Anderson et al. (2010). Briefly, founders from the HapMap CEU, CHB, JPT, and YRI ancestral populations were used to train the model Duan et al. (2008). Common SNPs between all four populations and the AGRE or SSC cohort were identified in plink. Alleles were forced to the same strand, A→T and G→C SNPs were excluded because of alignment concerns. The final dataset was analyzed utilizing EIGENSTRAT Price et al. (2006). Samples of European ancestry in the AGRE and SSC cohort were determined by deviation in PCA components 1 and 2 between the AGRE or SSC samples and the HapMap CEU individuals. In order to be classified as European, a cohort individual was required to have a PCA component 1 and PCA component 2 value within 1.5 standard deviations of the HapMap CEU PCA component 1 and 2 means.

4. Defining SNPs that escape X-inactivation

Regions that escape X inactivation were determined according to the results of a gene-based rodent/human somatic cell hybrid study Carrel and Willard (2005). Nine hybridomas were assessed. If gene expression was observed from an inactivated human X chromosome in at least three hybridomas, that gene was considered to escape X-inactivation. To identify regions, rather than genes, regions between consecutive escaping genes were also defined as escaping X inactivation. The co-ordinates used were defined by the first gene start position and last gene stop position plus 1kbp at either end to include regulatory regions in close proximity. UCSC gene definitions and hg18 genomic co-ordinates were used throughout (Figure 3 main manuscript and Table S4).

5. SRS based subject groupings of females in multiplex autism families

We considered the possibility that females with and without protection may be best defined by considering SRS scores instead, or in combination, with ASD affected and unaffected diagnosis. Our first exploratory analysis was based purely on parent SRS score and identified a high SRS 'case' group and a low SRS 'control' group (Figure S1); sample sizes are based on females with high quality data and European ancestry who were used in the analysis (Table S3):

- **High SRS** SRS >45; N=103 in AGRE; N=198 in SSC
- **Low SRS** SRS ≤45; N=74 in AGRE; N=621 in SSC

No SNPs reached significance after correcting for multiple comparisons in this analysis (Figure S5, S6, S7, S8, S9, S10 and Table S5).

Since AGRE is a multiplex collection, the majority of individuals are likely to carry a high burden of ASD genetic risk compared with the general population. To identify a subset of females likely to be enriched for the protective allele, we first classified females as being affected or unaffected, as in the analysis shown in the main manuscript. We then further stratified females within each diagnostic group by their SRS score to identify four cohorts (Figure S11); sample sizes are based on samples with high quality data and European ancestry who were used in the analysis:

- **Affected, high SRS** upper 50% of all affected females; N=58 in AGRE
- **Affected, low SRS** lower 50% of all affected females; N=51 in AGRE
- **Unaffected, high SRS** SRS >45; N=54 in AGRE
- **Unaffected, low SRS** SRS ≤45; N=64 in AGRE

We performed the following exploratory association analyses with alternative definitions of the protected and unprotected groupings of females:

- Affected, high SRS (unprotected) vs Affected, low SRS (protected)
- Affected, high SRS (unprotected) vs Unaffected, high SRS (protected)
- Affected, high SRS (unprotected) vs Unaffected, low SRS (protected)
- Affected, low SRS (unprotected) vs Unaffected, high SRS (protected)
- Affected, low SRS (unprotected) vs Unaffected, low SRS (protected)
- Unaffected, high SRS (unprotected) vs Unaffected, low SRS (protected)

None of these analyses produced SNPs with p-values exceeding the significance threshold after correction for multiple SNP comparisons (Table S5). Given the exploratory nature of this analysis we did not correct for the multiple association tests.

6. Assessing the impact of ascertainment bias on liability

The multiplex families used in AGRE for the initial observation of a bimodal SRS distribution in females were selected on the basis of having two children affected with ASD. To assess the impact of this ascertainment bias on the distribution of ASD liability we developed a simulation (R code shown below).

A male and female parent were assigned a random value for ASD liability based on a standard normal distribution (mean=0, SD=1). To model the female protective effect the mean liability in males was increased by 0.33 standard deviations and decreased by the same factor in females. These values produced a 4:1 sex bias. A diagnostic z-score threshold of 2.93 was set to produce an incidence of 1%.

The mean liability of the two parents was estimated and this mean was used to estimate a random value for the ASD liability of two male children and two female children. As with the parents, the male mean was increased by 0.33, while the female mean was decreased by the same factor. If two children in the family exceeded the diagnostic threshold the family was included and the liability estimates returned.

1 million families were simulated and the liability distribution of the male and female children were plotted (Figure S12A and S12B). The resulting distribution was made up of two overlapping distributions: 1) the tail end of the normal distribution cut-off at the diagnostic threshold representing the children that contributed to the ascertainment; and 2) a normal distribution with a mean above the population mean, but below the diagnostic threshold representing the children in ascertained families that did not contribute to ascertainment.

With the addition of noise, as would be expected using a proxy measure such as the SRS, the female distribution is likely to appear bimodal while the male would be unimodal.

However, this does not reproduce the initial observation of a bimodal SRS in multiplex females in two important respects:

1. For the lower distribution in females the mean is at least one standard deviation above the population (S12B), however for the SRS distribution in females the SRS scores were comparable to the normal population (Figure 1B).
2. The difference in male and female SRS distributions in the general population is 3 points, equivalent to 0.17 standard deviations. This is four-fold less than the difference in male and female mean used in the simulation

shown in Figures S12A and S12B. Using the 0.17 standard deviation value results in the distributions shown in Figure S12C and S12D in which the distributions are similar between the sexes.

Therefore, while ascertainment bias may partially explain the bimodal SRS in multiplex females it is far from a complete explanation of this phenomena. The R code used to perform this simulation is included below:

```
library(parallel)

# Mean/threshold selected to give 1% incidence with 4:1
    sex bias
fam <- function (val, m.mean=0, diff=0.33, pop.stdev=1,
    thres=2.93) {

        # Create parents and derive family mean
        father <- rnorm(1,m.mean + diff,pop.stdev)
        mother <- rnorm(1,m.mean - diff,pop.stdev)
        fam.mean <- mean(c(father,mother))

        # Create two male and two female kids based on
            family mean
        m.kids <- rnorm(2,fam.mean + diff,pop.stdev)
        f.kids <- rnorm(2,fam.mean - diff,pop.stdev)

        # Assess affected status
        m.affected <- length(which(m.kids >= thres))
        f.affected <- length(which(f.kids >= thres))

        # Only return liability for families with 2
            affected kids
        if (m.affected + f.affected >= 2) {
            return (list(m.kids, f.kids))
        } else {
            return (NA)
        }
}

# Create 1 million families
dist <- mclapply( 1:1000000, fam, mc.cores=4 )
```

```
# Remove 'NA's and split by sex
dist2 <- dist[!is.na(dist)]
m.dist3 <- unlist(lapply(dist2, "[[", 1))
f.dist3 <- unlist(lapply(dist2, "[[", 2))

# Plot male and female liability
hist(m.dist3, breaks=seq(-3,7,by=0.2), col="blue", prob=
   TRUE, main="Male distribution", xlab="ASD liability (
   z-score)")
lines(density(m.dist3, na.rm=TRUE), col="black", lwd=2)

hist(f.dist3, breaks=seq(-3,7,by=0.2), col="red", prob=
   TRUE, main="Female distribution", xlab="ASD liability
    (z-score)")
lines(density(f.dist3, na.rm=TRUE), col="black", lwd=2)
```

References

Anderson, C. A., Pettersson, F. H., Clarke, G. M., Cardon, L. R., Morris, A. P. and Zondervan, K. T. (2010), 'Data quality control in genetic case-control association studies.', *Nat Protoc* **5**(9), 1564–1573.

Anney, R., Klei, L., Pinto, D., Almeida, J., Bacchelli, E., Baird, G., Bolshakova, N., Bölte, S., Bolton, P. F., Bourgeron, T., Brennan, S., Brian, J., Casey, J., Conroy, J., Correia, C., Corsello, C., Crawford, E. L., de Jonge, M., Delorme, R., Duketis, E., Duque, F., Estes, A., Farrar, P., Fernandez, B. A., Folstein, S. E., Fombonne, E., Gilbert, J., Gillberg, C., Glessner, J. T., Green, A., Green, J., Guter, S. J., Heron, E. A., Holt, R., Howe, J. L., Hughes, G., Hus, V., Igliozzi, R., Jacob, S., Kenny, G. P., Kim, C., Kolevzon, A., Kustanovich, V., Lajonchere, C. M., Lamb, J. A., Law-Smith, M., Leboyer, M., Le Couteur, A., Leventhal, B. L., Liu, X. Q., Lombard, F., Lord, C., Lotspeich, L., Lund, S. C., Magalhaes, T. R., Mantoulan, C., McDougle, C. J., Melhem, N. M., Merikangas, A., Minshew, N. J., Mirza, G. K., Munson, J., Noakes, C., Nygren, G., Papanikolaou, K., Pagnamenta, A. T., Parrini, B., Paton, T., Pickles, A., Posey, D. J., Poustka, F., Ragoussis, J., Regan, R., Roberts, W., Roeder, K., Roge, B., Rutter, M. L., Schlitt, S., Shah, N., Sheffield, V. C., Soorya, L., Sousa, I., Stoppioni, V., Sykes, N., Tancredi, R., Thompson, A. P., Thomson, S., Tryfon, A., Tsiantis, J., Van Engeland, H., Vincent, J. B., Volkmar, F., Vorstman, J. A., Wallace, S., Wing, K., Wittemeyer, K., Wood, S., Zurawiecki, D., Zwaigenbaum, L., Bailey, A. J. et al. (2012), 'Individual common variants exert weak effects on the risk for autism spectrum disorderspi', *Hum Mol Genet* **21**(21), 4781–92.

Carrel, L. and Willard, H. F. (2005), 'X-inactivation profile reveals extensive variability in x-linked gene expression in females', *Nature* **434**(7031), 400–4.

Duan, S., Zhang, W., Cox, N. J. and Dolan, M. E. (2008), 'Fstsnp-hapmap3: a database of snps with high population differentiation for hapmap3.', *Bioinformation* **3**(3), 139–141.

Faul, F., Erdfelder, E., Lang, A.-G. and Buchner, A. (2007), 'G*power 3: a flexible statistical power analysis program for the social, behavioral, and biomedical sciences.', *Behav Res Methods* **39**(2), 175–191.

Fombonne, E. (2009), 'Epidemiology of pervasive developmental disorders.', *Pediatr Res* **65**(6), 591–8.

Price, A. L., Patterson, N. J., Plenge, R. M., Weinblatt, M. E., Shadick, N. A. and Reich, D. (2006), 'Principal components analysis corrects for stratification in genome-wide association studies.', *Nat Genet* **38**(8), 904–909.
